# Sampling-based Bayesian approaches reveal the importance of quasi-bistable behavior in cellular decision processes on the example of the MAPK signaling pathway in PC-12 cell lines

**DOI:** 10.1186/s12918-017-0392-6

**Published:** 2017-01-25

**Authors:** Antje Jensch, Caterina Thomaseth, Nicole E. Radde

**Affiliations:** Institute for Systems Theory and Automatic Control, University of Stuttgart, Pfaffenwaldring 9, Stuttgart, 70569 Germany

**Keywords:** Quasi-bistability, MAPK signaling pathway, Cellular decision making

## Abstract

**Background:**

Positive and negative feedback loops are ubiquitous motifs in biochemical signaling pathways. The mitogen-activated protein kinase (MAPK) pathway module is part of many distinct signaling networks and comprises several of these motifs, whose functioning depends on the cell line at hand and on the particular context.

The maintainance of specificity of the response of the MAPK module to distinct stimuli has become a key paradigm especially in PC-12 cells, where the same module leads to different cell fates, depending on the stimulating growth factor.

This cell fate is regulated by differences in the ERK (MAPK) activation profile, which shows a transient response upon stimulation with EGF, while the response is sustained in case of NGF. This behavior was explained by different effective network topologies. It is widely believed that this sustained response requires a bistable system.

**Results:**

In this study we present a sampling-based Bayesian model analysis on a dataset, in which PC-12 cells have been stimulated with different growth factors. This is combined with novel analysis methods to investigate the role of feedback interconnections to shape ERK response. Results strongly suggest that, besides bistability, an additional effect called quasi-bistability can contribute to explain the observed responses of the system to different stimuli. Quasi-bistability is the ability of a monostable system to maintain two distinct states over a long time period upon a transient signal, which is also related to positive feedback, but cannot be detected by standard steady state analysis methods.

**Conclusions:**

Although applied on a specific example, our framework is generic enough to be also relevant for other regulatory network modeling studies that comprise positive feedback to explain cellular decision making processes. Overall, this study advices to focus not only on steady states, but also to take transient behavior into account in the analysis.

**Electronic supplementary material:**

The online version of this article (doi:10.1186/s12918-017-0392-6) contains supplementary material, which is available to authorized users.

## Background

Feedback regulations are ubiquitous network motifs in all kinds of molecular interaction networks, such as for example metabolic networks, regulatory modules or signaling networks [1]. The role of single positive and negative feedback is well-characterized also from a theoretical point of view. Negative feedback, which counteracts external perturbations, can cause oscillating behavior, but also has a stabilizing effect, implies robustness of cell states to internal and external perturbations [2], and plays a major role in maintaining homeostasis (see e.g. [3–5]). Furthermore, it can accelerate the response to a transient signal. By contrast, positive feedback amplifies an external perturbation or signal, which can cause multi-stability, hysteresis and memory effects or switch-like behavior. Positive feedback is omnipresent in cellular decision processes, in which these phenomena arise. It can also produce ultrasensitivity and prolong the response to a transient external signal [5–7].

In this study we investigate the role of feedback regulation for proper signal processing by a case study on the well-known mitogen-activated protein kinase (MAPK) signaling pathway. This pathway is an evolutionary conserved signaling module, which is involved in many essential cellular processes such as proliferation, survival or differentiation [8–11]. It is de-regulated in various diseases and represents an important drug target [11]. The pathway module consists of a cascade of phosphorylation events, leading to the activation of ERK, which targets more than 80 substrates in the nucleus and the cytosol. It is integrated into multiple signaling pathways and shows a variety of different responses depending on the stimulus and the cell-type specific context [9, 11, 12]. Specificity of the cellular response is tightly related to distinct time courses of active ERK upon different stimuli, in particular amplitude and duration of the signal response [11–13]. A well-studied paradigm for such a context-specific response is the different behaviors of PC-12 cells upon stimulation with epidermal growth factors (EGF) and neural growth factors (NGF) [12, 14]. Cells stimulated with NGF show sustained activation of ERK, accompanied by a translocation of ERK into the nucleus, which eventually initiates cell differentiation. In contrast, ERK activity is transient and mainly restricted to the cytosol upon stimulation with EGF, which in turn triggers proliferation.

The pathway module is well-characterized experimentally and from a modeling point of view (for reviews see e.g. [9–11, 15–17]). Starting with the early work of Huang and Ferrell [18], many models of different complexity and with different foci have been suggested in the meantime [11, 19–22]. In particular, quite a number of studies focus on modeling and understanding the mechanisms behind the distinct responses upon EGF and NGF stimulation in PC-12 cells [12–14, 23–26].

It is commonly believed and well-described that a system which shows a sustained response to a transient signal, such as PC-12 cells upon NGF stimulation, is a bistable system [15, 19, 23, 25–30]. Hence modeling of this phenomenon usually focuses on the investigation of the bistability properties of respective models, and advanced methods have been developed tailored to the investigation of steady states in these models (see e.g. [23, 31, 32]).

In this study we turn our attention to a phenomenon called quasi-bistability and its role in the regulation of the MAPK module for cellular decision making. Quasi-bistability is the ability of a monostable system to maintain a second steady state for a long period of time upon a transient stimulus [33]. It is also related to positive feedback, but less well investigated and understood. Using a dynamic modeling approach and a dataset of the MAPK module in PC-12 cell lines, the system is analyzed via Bayesian sampling techniques. Mechanisms behind sustained ERK responses are investigated by a combination of steady state analysis methods and novel methods that also allow to investigate time scales of transient behavior.

## Methods

### Experimental data used for model calibration

**Fig. 1:**
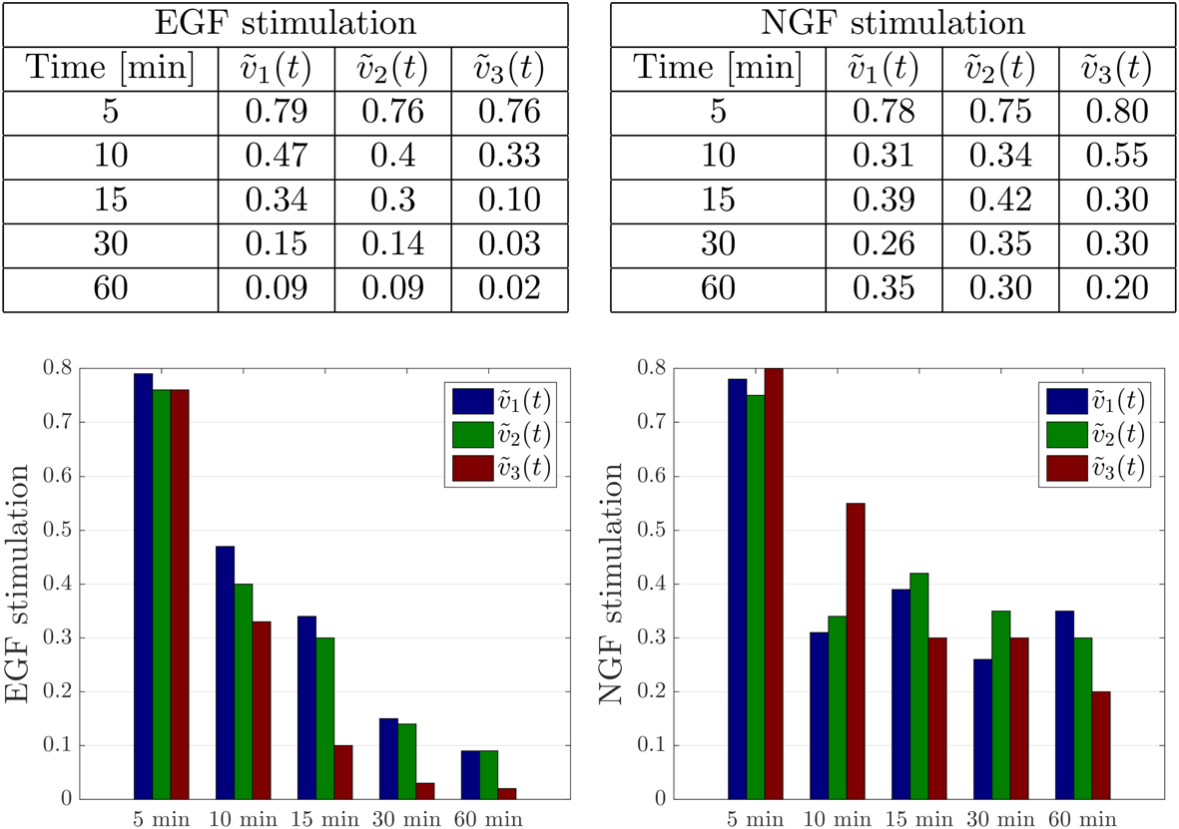
Activities of Raf, MEK and ERK after stimulation. Scaled activities of Raf, MEK and ERK measured by polychromatic flow cytometry (by visual inspection from Fig. S1b in [12])

For our modeling study we used a dataset described in [12], where PC-12 cell lines were stimulated with EGF and NGF, and phosphorylation of the proteins in the cascade was measured via Western blotting and flow cytometry. For model calibration we used the data shown in Figs. 1 and S1b in [12]. This dataset contains data from control experiments, in which cells were stimulated with 100 ng/ml EGF or 50 ng/ml NGF, and measurements from RNA interference experiments.

In the control experiments the dynamic response of the system upon stimulation was measured in terms of phosphorylation levels of Raf (pRaf), MEK (ppMEK) and ERK (ppERK). In the following we will refer to the active states of the proteins by using the following variables:

$$\begin{array}{*{20}l} v_{1} &= \text{pRaf}\\ v_{2} &= \text{ppMEK}\\ v_{3} &= \text{ppERK}. \end{array} $$

We used data from flow cytometry experiments (Fig. S1b in [12]) as reference for model calibration, since all proteins were quantified in this experiment. Extracted values are illustrated in Fig. 1 and show a transient signal response in case of stimulation with EGF, and a sustained response after stimulation with NGF. The quantified values are a scaled version of the quantities *v*_*i*_, and are defined as $\tilde v_{i}$.

In the siRNA experiments, Raf, MEK and ERK were consecutively downregulated. These data were used in [12] to analyze the network topology via Modular Response Analysis [34]. In this analysis, global response coefficients *R*_*ij*_, *i*,*j*=1,2,3 were calculated from the Western blot signals (Fig. S1c in [12]) via

1 $$ R_{ij} = 2\frac{\partial \ln(v_{i})}{\partial \ln(p_{j})}\approx 2\frac{\bar v_{i}^{(s_{j})}-\bar v_{i}^{(c)}}{\bar v_{i}^{(s_{j})}+\bar v_{i}^{(c)}}.   $$

The variables $\bar v_{i}^{(c)}$ and $\bar v_{i}^{(s_{j})}$ denote the steady state concentrations of variable *v*_*i*_ before and after perturbation *p*_*j*_, i.e. silencing of component *j*, respectively.

Equation (1) can be resolved for ${\bar v_{i}^{(s_{j})}}/{\bar v_{i}^{(c)}}$,

2 $$ \frac{\bar v_{i}^{(s_{j})}}{\bar v_{i}^{(c)}} = \frac{2+R_{ij}}{2-R_{ij}},  $$

which gives the concentration change of component *i* relative to the control experiment in response to silencing of component *j*.

**Fig. 2:**
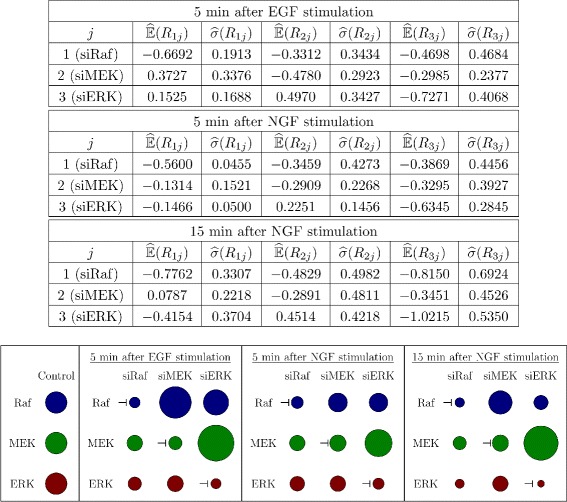
Data from modular response analysis. Table. Means and standard deviations of the global response coefficients extracted from the silencing experiments via modular response analysis. These were calculated from replicates in Table S1d in [12]. Figure. Illustration of respective changes in protein concentrations in response to silencing relative to the control experiments (without silencing)

Values of the response coefficients of four replicates for silencing of each protein are provided in Table 1 in Fig. S1d in [12]. These data were used to calculate empirical means and standard deviations, as illustrated in Fig. 2, together with the respective relative changes of protein concentrations after silencing.

Time points were set to 5 min after EGF stimulation, the time about which the maximum of the signal response is reached in the control experiments, which is assumed to be close to a steady state condition. In case of NGF, global response coefficients are given at 5 and 15 min after stimulation. These two time points correspond to the times at which the maximum of the signal response was reached and at which the system seems to have reached the new steady state. In [12], these coefficients were used to extract the network structure based on the so-called local response coefficients. This analysis indicates a positive feedback from ERK to Raf upon NGF stimulation and a negative feedback when stimulated with EGF. This result will be taken into account in our modeling approach.

### Sampling-based Bayesian approach for model calibration

#### Data-driven modeling approach

**Fig. 3:**
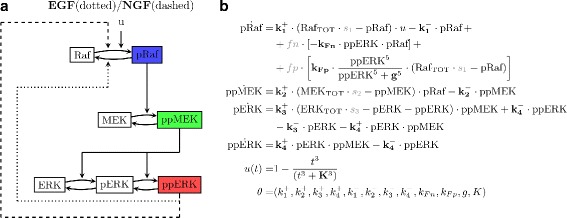
Model structure of the MAPK module. **a** Reaction scheme of the MAPK module. Upon addition of growth factors, Raf, MEK and ERK are successively activated in a phosphorylation cascade. Different feedback topologies are assumed to shape context dependent ERK response: Effective negative feedback from ERK to Raf upon EGF stimulation (*dotted line* from ppERK to dephosphorylation of pRaf), and positive feedback in case of NGF stimulation (*dashed line* from ppERK to phosphorylation of Raf). **b** Differential equation model of the MAPK cascade. Bold parameters are the unknown constants, collected in the parameter vector *θ*, while gray parameters define the specific experimental condition for the simulation

Based on the experimental data available for model calibration and on existing modeling studies for the MAPK module [10, 18, 23], we formulated a differential equation model based on mass action kinetics for the three-tiered phosphorylation cascade (Fig. 3).

In this cascade, both MEK and ERK require dual phosphorylation to become fully active. Double phosphorylation makes the cascade behave in an ultrasensitive way, which is advantageous for noise filtering [9, 11, 35]. MEK phosphorylation is processive, i.e. both sites are phosphorylated in a single step, whereas ERK phosphorylation is distributive and requires two interactions [9, 35]. We have taken this into account by modeling MEK double phosphorylation as a single reaction, while full activation of ERK is obtained in a two step reaction.

Furthermore, we exploited conservation of total protein concentrations,

3a $$\begin{array}{*{20}l} \text{Raf}_{\text{TOT}} \cdot s_{1} &= \text{Raf} + \text{pRaf} \end{array} $$

3b $$\begin{array}{*{20}l} \text{MEK}_{\text{TOT}} \cdot s_{2} &= \text{MEK} + \text{ppMEK} \end{array} $$

3c $$\begin{array}{*{20}l} \text{ERK}_{\text{TOT}} \cdot s_{3} &= \text{ERK} + \text{pERK} + \text{ppERK} \end{array} $$

to end up with a four variable model, shown in Fig. 3 b. Rate constants are denoted by $k_{i}^{+/-}$, *i*=1,…,4, reduction of total protein amounts in the siRNA perturbation experiments are described by the silencing factors *s*_*i*_∈(0,1] (*i*=1,2,3). These factors were extracted from quantification of the proteins in the control and the silencing experiments, as reported in Fig. 1c of [12]. Their values were set to *s*_1_=0.72, *s*_2_=0.7 and *s*_3_=0.65 when simulating silencing of Raf, MEK or ERK, respectively.

The input *u*(*t*), which mimics signal initiation after addition of growth factor and summarizes all upstream processes, was described via a sigmoidally decreasing function, whose parameter *K* was also included in the optimization procedure,

4 $$ u(t) = \left\lbrace \begin{array}{ll} 0 & t < 0\\ 1-\frac{t^{3}}{t^{3}+K^{3}} & t \ge 0. \end{array}\right.  $$

Thus, *u*(*t*) jumps from 0 to 1 at time *t*=0, which mimics addition of ligand, and subsequently decreases sigmoidally, reflecting observations of transient Ras activity, which is upstream of Raf and returns to its inactive Ras-GDP state within five minutes [19]. This implies that our model has a trivial steady state in which all variables are equal to 0 for *u*=0, which is also a simplification, since proteins usually have minimal basal activities. However, since we do not have data for *t*=0, which would reflect these basal activities, and since these are anyway assumed to be very low compared to the stimulated case [36], we consider this simplification not a crucial one.

In our model the input *u*(*t*) is not directly coupled to the network structure, which is clearly a simplification, since EGF and NGF trigger different receptor systems. However, exactly the same model structure has been used in other studies as well (see e.g. [27]) and was shown to display a rich variety of different behaviors, including ultrasensitivity and bistability and, as we will demonstrate, is also sufficient to capture various observed responses. Moreover, we follow here the argumentation in [14], according to which the different ERK responses are unlikely to be caused by different receptor systems. The Boolean variables *f*_*p*_ and *f*_*n*_ account for the experimental condition and act as switches between the two network structures, depending on the growth factor.

The positive feedback from ERK to Raf that was postulated from the modular response analysis in [12] was described by a sigmoidal function in order to facilitate bistability. Although this feedback is not necessarily required for bistability in the MAPK signaling pathway [21, 28–30], it has been shown to enhance the range of bistable behavior and to make the occurrence of bistability less sensitive to stochastic fluctuations and parameter variations [30].

#### Model calibration procedure

In the next step we inferred the unknown model parameters

5 $$ \theta=\left(k_{1}^{+}, k_{2}^{+}, k_{3}^{+}, k_{4}^{+}, k_{1}^{-}, k_{2}^{-}, k_{3}^{-}, k_{4}^{-}, k_{Fn}, k_{Fp}, g, K\right)  $$

by using the described set of data *y*. For this model calibration procedure we used a sampling-based Bayesian approach, which provides a consistent statistical description for all quantities-of-interest. In a Bayesian approach, parameters *θ* and measurements *y* are interpreted as random variables that are characterized by probability distributions. Hence such an approach offers full information about uncertainties in terms of underlying distributions. A short explanation of the Bayesian idea is provided in Additional file 1.

In our Bayesian framework the ODE model is stochastically embedded by defining the underlying stochastic process from which the experimental data are assumed to be generated. This is sometimes also referred to as noise model (see Additional file 1 for more details). Here we exploit log-normal error models for protein concentrations, using the same standard deviation of 0.2 for the logarithmic transformation of the experimental data, which by definition are normally distributed.

These are translated into respective error models for the global response coefficients via transformation of probability distributions. Altogether, this defines the likelihood function *l*_*y*_(*θ*)=*p*(*y*|*θ*), which is a measure of how likely it is to see the experimental data given a particular model.

In a Bayesian framework, the objective function of interest is the posterior distribution *p*(*θ*|*y*), which is a distribution of parameters conditional on the given dataset. According to the Bayes Theorem, the posterior distribution is proportional to the product of the prior distribution *p*(*θ*) of the parameters and of the likelihood function,

6 $$ p(\theta|y) = \frac{p(y|\theta)p(\theta)}{p(y)}.  $$

Since the light signals of the Western blot data require appropriate rescaling and normalization to a reference experiment for a comparison across different experimental conditions, the ODE model in Fig. 3 b also had to be rescaled and normalized in order to enable a comparison with these data. This procedure is described in Additional file 2. Moreover, a detailed formulation of the posterior distribution is given in Additional file 3.

We investigate the posterior distribution by generating samples {*θ*_*i*_}_*i*=1,…,*N*_ via Markov Chain Monte Carlo (MCMC) sampling. These samples are subsequently used for Monte Carlo estimates of other quantities-of-interest. For example, the posterior predictive distribution (PPD) to see new data $\tilde y$ in any experimental scenario is given by

$$\begin{array}{*{20}l} {}p(\tilde y|y) &= \int_{\Theta} p(\theta,\tilde y|y)d\theta \quad \text{Marginalization}\\ &= \int_{\Theta} p(\tilde y|\theta,y)p(\theta|y)d\theta \quad \text{Factorization}\\ &= \int_{\Theta} p(\tilde y|\theta)p(\theta|y)d\theta\!\! \quad \tilde y \text{ is independent of } y \text{ given } \theta \\ &\approx \! \frac{1}{N}\sum_{i=1}^{N} p(\tilde y|\theta_{i})\!\! \quad \theta_{i} \! \sim \! p(\theta|y)\!\! \quad \text{Monte Carlo estimate} \end{array} $$

If not stated otherwise, model predictions are consistently given in terms of these PPDs in this work.

## Results

### Calibrated model describes experimental data

We generated samples {*θ*_*i*_}_*i*=1,…,*N*_ from the posterior distribution as described (see also Additional file 4 for implementation details). Kernel density estimates of the marginal parameter distributions and 2D scatter plots for the two-dimensional parameter marginals are shown in Additional files 5 and 6. Most of the parameters show a large variance. The only exceptions are the dephosphorylation rates of pRaf and ppMEK, which mainly determine the speed of the decay of the signal. Moreover, the threshold parameter *K* of the input signal can be extracted from the data. There are also almost no correlations visible in the 2D scatter plots except a strong positive correlation between *k*_*Fp*_ and $k^{+}_{1}$.

**Fig. 4:**
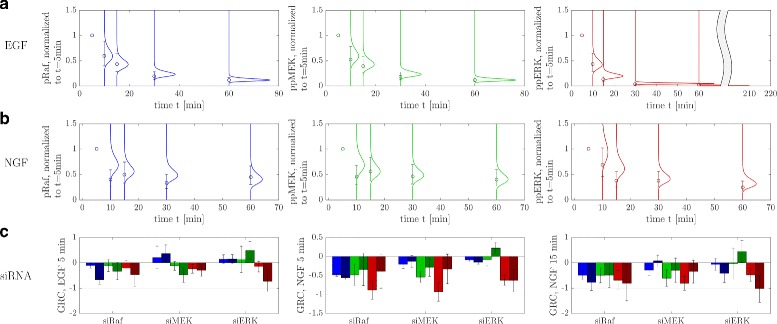
Calibrated model using a Bayesian approach. Dynamic responses of pRaf, ppMEK and ppERK after stimulation with EGF **a** and NGF **b**. Shown are the values acquired from flow cytometry experiments (Fig. 1) in comparison to the respective PPDs predicted by the model. Data have been normalized to *t*=5 min. **c** Comparison of data for the global response coefficients (GRC) extracted from the siRNA perturbation experiments (Fig. 2), and respective simulated distributions, here for clarity represented with the first and second moment. Data are taken from [12]

Figure 4 shows the result of the Bayesian model calibration in the prediction space. Depicted are the Monte Carlo estimates of the PPDs in comparison with experimental data. Figure 4 a and b show the dynamic responses of the observables pRaf, ppMEK and ppERK in the control experiments after stimulation with EGF (A) and NGF (B). The model captures the EGF scenario very well, with low variances in the PPDs. In case of NGF some data points are slightly overestimated, but the data are still within the predicted confidence intervals, which are larger here compared to the EGF scenario. The colors chosen for pRaf (blue), ppMEK (green) and ppERK (red) are maintained for all simulation results throughout the paper.

A comparison of the global response coefficients is depicted at the bottom (Fig. 4 c). The sign structure is preserved for almost all silencing experiments, the only exception being MEK in the siERK experiments with NGF stimulation. This is due to the fact that we did not include the direct negative feedback from ERK to MEK that was postulated from the modular response analysis in [12] in our model, since the signal to noise ratio was rather low for this interaction, and we wanted to keep the model simple. At first glance the fits seem to be reasonable, which is however hard to judge solely from visual inspection, since error bars are large for most of these values. This is also mirrored by the variances of the PPDs. Thus we decided to validate the model via predictions of further experiments with the same cell line that were not used for model calibration.

### Model is able to predict various perturbation experiments

**Fig. 5:**
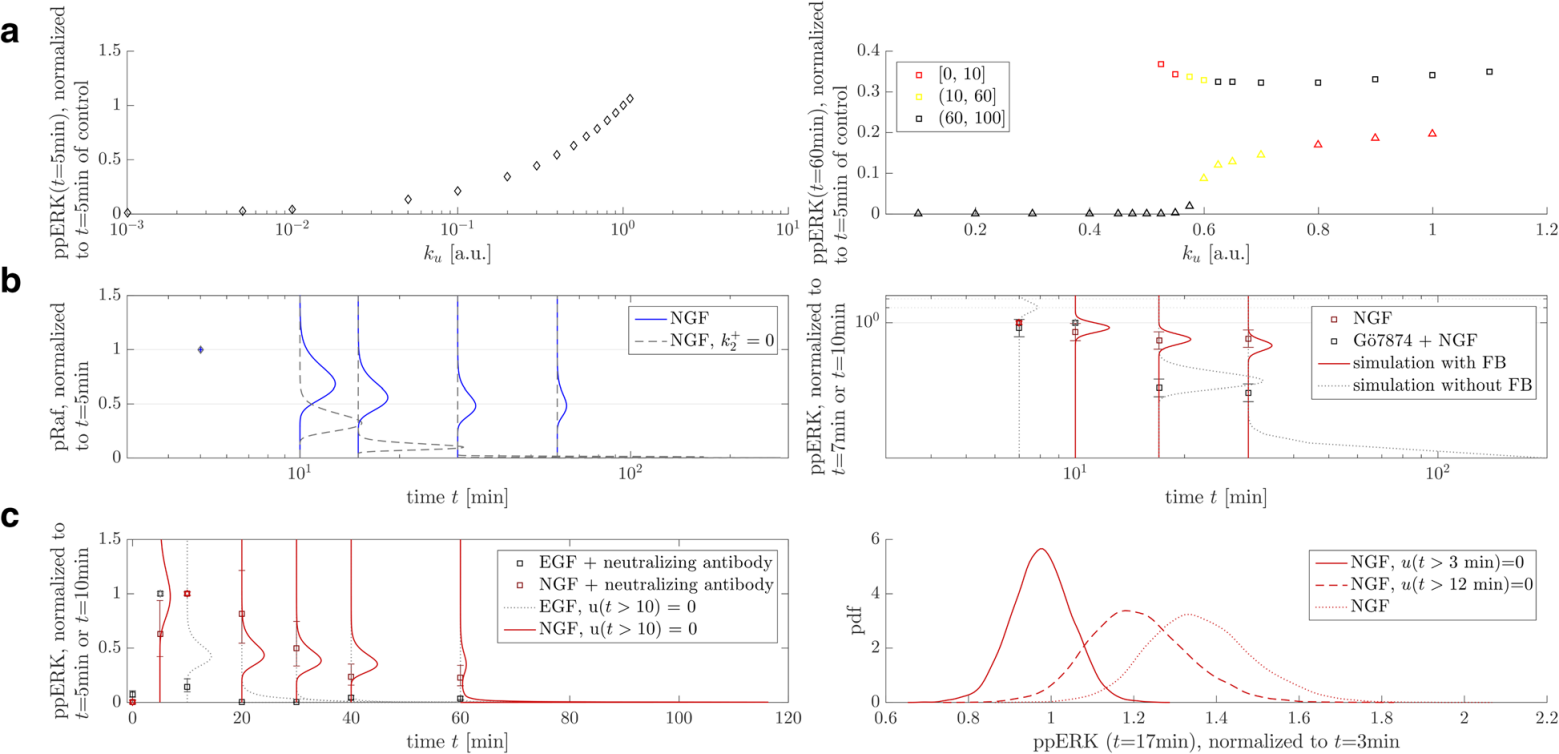
Model validation. **a** Dose-response profiles of ERK activation were mimicked by simulating the model with increasing input strength parameter *k*_*u*_ for stimulation with EGF (*left*) and NGF (*right*). The system shows a unimodal and ultrasensitively increasing ppERK concentration after stimulation with EGF (*t*=5 min after stimulation) and a bimodal distribution when stimulated with NGF exceeding a threshold concentration (*t*=60 min after stimulation) (compare data in [12], Subfigs 2c and d). **b** Inhibition of MEK (*left*) results in the loss of sustained Raf activation upon stimulation with NGF (gray dashed PPDs) compared to the control case (blue continuous PPDs). Inhibition of PKC via Gö7874 (*right*) causes the loss of sustained ERK activation upon NGF stimulation (data from [12], Fig. 4a). This was simulated by switching off the feedback connection. **c** Irreveversibility in MAPK activation upon NGF stimulation was investigated via mimicking treatment of the cell culture with neutralizing antibodies (*left*) and TrkA inhibitors (right) (compare data in [12], Subfigs 3a and c)

For model validation we decided to use the model to predict outcomes of a set of perturbation experiments that have not been used for model calibration. The result is shown in Fig. 5. In particular, the following experimental setups were considered:

#### Dose response profiles of ERK activation

We mimicked dose-response profiles of ERK activation to increasing EGF and NGF doses measured via flow cytometry (Fig. 2 in [12]). Since these datasets are single-cell measurements that represent a heterogeneous cell population, we interpreted our parameter samples to represent such a cell population, whose average is consistent with the data used for calibration, and whose distribution accounts for population heterogeneity. Increasing ligand concentration was reflected by multiplying the parameter $k_{1}^{+}$, which describes the input strength, by a factor *k*_*u*_. Resulting mean values of ppERK are shown in Fig. 5 a. We note here that this comparison can only be done in a qualitative way, since we lack a receptor model that directly relates growth factor concentrations to the input signal for Raf activation. Thus, it is here not possible to include the respective experimental data directly for comparison. However, model simulations capture the observed qualitative phenomena quite well: In case of stimulation with EGF the ERK activity profile is unimodal and raises sigmoidally with increasing EGF concentration. In contrast, upon stimulation with NGF the profile becomes bimodal when NGF doses exceed a threshold. Moreover, with increasing NGF concentrations the fraction of cells with a sustained response as well as the mean ERK activities of both subpopulations increase.

#### Effect of feedback breaking via inhibition of MEK and PKC

We predicted the influence of MEK inhibition via the MEK inhibitor PD184352 on the temporal activity of Raf (Fig. S1e in [12]), by assuming that MEK activation is completely abolished. This was realized in our model by setting the MEK phosphorylation rate $k_{2}^{+}$ to zero, which destroys the feedback from ERK to Raf in the simulations, and inspection of Raf activity (Fig. 5 b left). While the response is sustained in the control case (blue continuous PPDs), MEK inhibition results in the loss of sustained Raf activity, and pRaf follows the transient signal and rapidly drops within a few minutes (gray dashed PPDs). This result is in agreement with the observations in [12].

In addition, we mimicked the inhibition of PKC via Gö7874 during NGF stimulation (Fig. 4a in [12]). We considered the feedback to be completely eliminated as a result and realized this by removing the feedback connections from our model (Fig. 5 b right). In the control case (red continuous PPDs) activity of ppERK was sustained, whereas the feedback deletion caused a decrease in ERK activation (gray dotted PPDs), again in accordance with experimental findings.

#### Irreversibility in MAPK activation

Finally, we also compared our model to experimental data on the irreversibility in MAPK network activation upon NGF stimulation, which was investigated via terminating the signal by growth factor neutralizing antibodies and TrkA inhibitors (Subfigs 3a and c in [12]). Therefore, both perturbations, i.e. addition of neutralizing antibody and TrkA inhibitor after stimulation, were mimicked via abrupt signal termination at the respective time points. Results are shown in Fig. 5 c. While in case of stimulation with EGF, ppERK was virtually zero shortly after addition of the neutralizing antibody (gray dotted PPDs in the left Figure), the NGF inhibition profile still showed some activity after 60 min (red PPDs).

For a further comparison we simulated ppERK time courses upon stimulation with NGF and addition of TrkA inhibitor at two different time points (Fig. 5 c right). PPDs for ppERK are depicted at *t*=17 min after stimulation when TrkA inhibitor was given at *t*=3 min after stimulation (continuous curve) and *t*=12 min after stimulation (dashed curve), compared to the control case (dotted curve). In agreement with experimental findings, results show that ERK activity rapidly drops in case that the stimulus terminated too early.

Overall, the results in Fig. 5 nicely demonstrate that our model is able to predict many important features of the signaling cascade quite accurately. Since these simulation scenarios capture the responses of the system to several treatments that are quite different from the experiments which have been used for fitting, the model is validated to have predictive power.

In the next step we decided to use the model to analyze mechanisms behind sustained ERK response in case of NGF stimulation.

### Mechanism behind sustained response caused by NGF

#### Bifurcation analysis reveals that bistability is not sufficient to explain model outcomes upon NGF stimulation

**Fig. 6:**
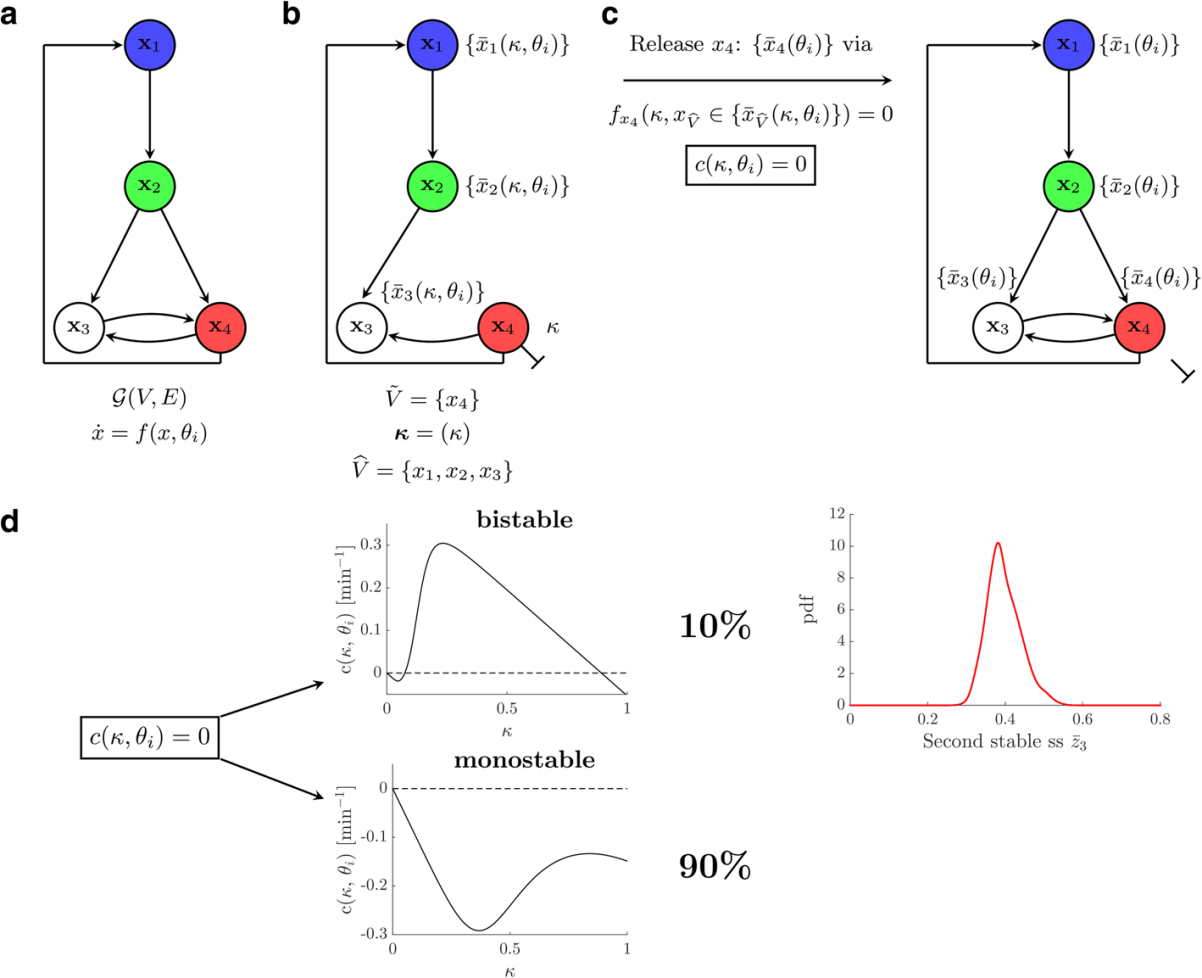
Steady state analysis using the circuit-breaking algorithm. The CBA is used for an efficient calculation of the steady states of the system for the MCMC parameter samples and subsequent automatic classification into mono- and bistable systems (**a-c**). **d** Result of this classification analysis. Depicted is also the distribution of the second stable steady state $\bar z_{3}\ne 0$ in case of a bistable system, which corresponds to the concentration of active ERK normalized to *t*=5 min (see Additional file 2)

In order to investigate the mechanisms behind sustained response to transient NGF signals, we combined our sampling-approach with the circuit-breaking algorithm (CBA) [32], which allows for an efficient calculation of steady states based on the topology of the signaling network, and for an automatic classification into mono- and bistable systems. Our approach is schematically illustrated in Fig. 6. Figure 6 a-c illustrate the steps of the CBA applied to our network model for a single parameter sample *θ*_*i*_. The CBA operates on the topology of the interaction graph *G*(*V*,*E*), which is a directed graph that shows dependencies between variables in the model (Fig. 6 a). In the first step all feedback loops^1^ are broken by deleting incoming edges for a suitably chosen subset $\tilde V$ of vertices and setting the respective variables to fixed values *κ*. The remaining vertices are collected in the set $\widehat V$. Here we set $\tilde V=\lbrace x_{4}\rbrace $, *x*_4_=*κ* and $\widehat V=\lbrace x_{1},x_{2},x_{3}\rbrace $. The state variables *x*_*i*_,*i*=1,…,4, in the interaction graph refer to the rescaled states of our ODE model that we used for all simulations (see Additional file 2). Then we calculated the steady state coordinates of the variables in $\widehat V$ in dependence of the input *κ*, obtaining the set $\bar x_{\widehat V}(\kappa,\theta _{i})$ (Fig. 6 b). In the last step the circuits are released one after another by releasing vertices in the set $\tilde V$ (Fig. 6 c). Mathematically, this translates here into the calculation of the zeros of the circuit-characteristic *c*(*κ*,*θ*_*i*_), which is given by

7 $$ c(\kappa,\theta_{i})= f_{x_{4}}(x_{4}=\kappa, x_{\widehat V}\in \lbrace \bar x_{\widehat V}(\kappa,\theta_{i}) \rbrace)=0.   $$

The obtained zeros $\bar {\kappa }$ of the circuit-characteristic correspond to the steady state coordinates of the state variable *x*_4_, from which the set of steady states of the full system can be derived. All details about the calculation of the values for $\bar {\kappa }$ and of the expressions of the steady state coordinates for the other three state values $\bar {x}_{\widehat V}(\kappa,\theta _{i})$, as functions of the parameter sample, are given in Additional file 7.

We applied the CBA to all parameters of the estimated posterior sample. The outcome was automatically classified, by using this analysis, according to the number of steady states of the system (Fig. 6 d). Results show an overall probability of 10% for the system to be bistable. We found this a surprisingly small number, which indicates that bistability is probably not the main mechanism behind the observed sustained ERK activation. Even worse, our analysis only provides an upper bound in two respects: First, depending on the parameters *θ*_*i*_, not all trajectories of bistable systems might be pushed to the basin of attraction of the second fixed point by the transient signal. Second, this set might also contain bistable systems in which the distance of the two steady states is rather small, such that the bistability will not be visible in any real experiment. Furthermore, we simulated the ODE model with the obtained subset of parameter samples *θ*_*i*_ leading to a bistable system, and we calculated the distribution of the second positive stable steady state $\bar {x}_{4}$. This is shown in Fig. 6 d on the right, by considering the normalized state variable (see Additional file 2).

$$z_{3}(t) = x_{4}(t)/x_{4}(t=5\text{~min}). $$

Overall, this analysis suggests that bistability is not sufficient to explain the observed sustained activation of ERK after NGF stimulation.

#### Quasi-bistability can explain sustained ERK activation

**Fig. 7:**
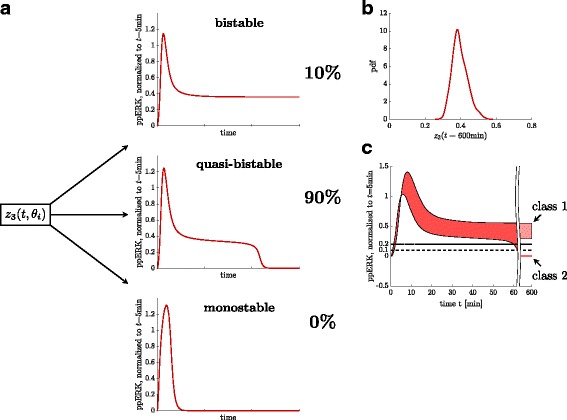
Simulation-based analysis of the long-term behavior of the cell population. **a** Trajectories are automatically classified into bistable, quasi-bistable and monostable systems, as described in the text. For stimulation with NGF in the control experiment 90% show a quasi-bistable behavior, while only 10% are really bistable. **b** Distribution of the second stable steady state distinct from zero of the bistable trajectories. **c** For the choice of threshold parameters that were used for the classification scheme we evaluated the 0% and 100% percentiles of ERK trajectories in the simulation of the NGF control experiment

We complemented our steady state analysis by a simulation-based classification of model trajectories after NGF stimulation, as illustrated in Fig. 7. A similar classification approach was used in [21], without explicitly investigating quasi-bistability.

We used the posterior sample to simulate model responses up to *t*=600 min. These responses were automatically classified in a second step (Fig. 7 a): Using ERK activity at *t*=5 min as a reference value, samples were sorted according to the following classification scheme:

- Class 1 (Bistable systems):
$\frac {\text {ppERK(60~min)}}{\text {ppERK(5~min)}}> 0.2$ and $\frac {\text {ppERK(600~min)}}{\text {ppERK(5~min)}}\ge 0.1$
- Class 2 (Quasi-bistable systems):
$\frac {\text {ppERK(60~min)}}{\text {ppERK(5~min)}}> 0.2$ and $\frac {\text {ppERK(600~min)}}{\text {ppERK(5~min)}}< 0.1$
- Class 3 (Monostable systems):
$\frac {\text {ppERK(60~min)}}{\text {ppERK(5~min)}}\le 0.2$

The threshold 0.2 for *t*=60 min was chosen such that all trajectories are above this value. This implies that class 3 is empty and no trajectory is classified to be simply monostable, as visualized in Fig. 7 c. This implication is reasonable, since *u*(*t*) is close to 0 already a few minutes after stimulation, and hence we expect simple monostable systems to follow the input with a delay that is much smaller than 1h. Figure 7 c also shows that the classification result is rather insensitive to fine-tuning of the second threshold at *t*=600 min: At this late time point the quasi-bistable and bistable trajectories are already well separated and there is a clear gap between the trajectories of classes 1 and 2.

The analysis revealed a fraction of 10% belonging to class 1. The estimated distribution of the second steady state equals that from the CBA analysis, which hints to the fact that trajectories of virtually all bistable systems detected via the CBA converge to the second steady state after stimulation with NGF. The rest of the samples, which are 90%, belong to class 2, which represent monostable systems that can show a sustained response for more than 60 min. after stimulation. However, trajectories in this class converge to their unique steady state at a later time point.

**Fig. 8:**
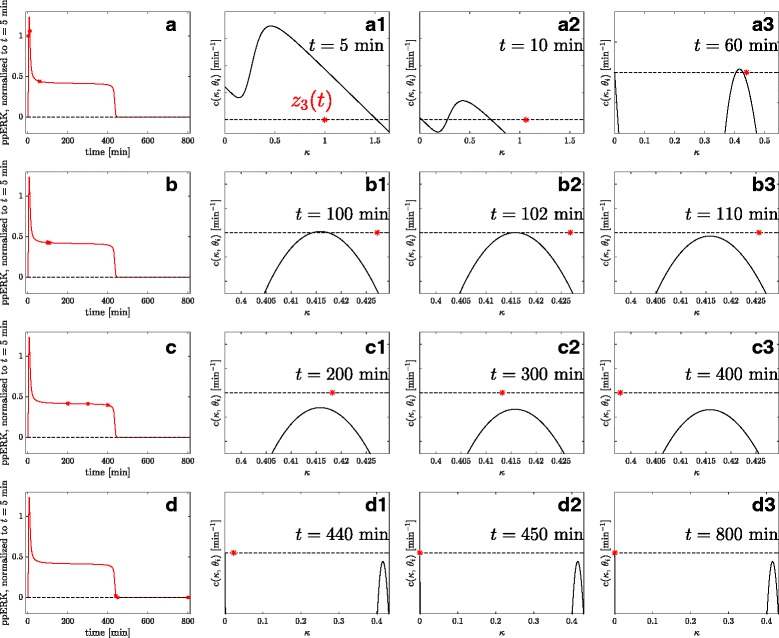
Quasi-bistability phenomenon. The CBA is used for the investigation of the quasi-bistability phenomenon, in which the system, despite being monostable, shows a very prolonged sustained response. The first column shows the time course of normalized ppERK for a representative parameter sample from class 2 with switching time at *t*_switch_≈440 min. Columns 2,3 and 4 show the circuit-characteristic *c*(*κ*,*u*(*t*)), along with the actual normalized state ppERK(t) for 12 different time points. After a fast transient dynamic (**a1**) the circuit-characteristic has three zeros (A2-B1), which disappear at a later time point, here *t*=102 min (**b2**), via a saddle-node bifurcation. After 60 min the input is almost zero and the vector field and therefore the circuit-characteristic changes only slowly. The system state has almost approached the higher fixed point. **b1**-**c3** are eyeglass views on the dynamics near this second fixed point. These plots show that, even if the fixed point has disappeared, the system trajectory moves very slowly through the state space for a rather long time, since $\dot {x}$ is still small. Only after about 440 min the system has overcome this slow region of the state space, and from here on rapidly moves towards its globally asymptotically stable steady state $\bar {x}=0$

In order to understand the mechanism behind this highly prolonged response to a transient input signal, we filtered the parameter sample for monostable systems that belong to class 2 and investigated their behavior in more detail. Therefore, we used the input *u*(*t*) as a bifurcation parameter and investigated the respective time-varying set $\lbrace \bar x(u)\rbrace $ of steady states of the system via the CBA. Figure 8 shows the temporal behavior of the set $\lbrace \bar z_{3}(u(t))\rbrace $ for a representative parameter sample belonging to class 2. After a fast transient phase, the system is bistable, since *u*(*t*) is sufficiently large to maintain two stable steady states. However, the second stable steady state vanishes due to a rapidly decreasing *u*(*t*). For the trajectory at hand the system becomes monostable already at about *t*≈102 min, which is fast compared to its switching time at *t*≈440 min. This comes from the fact that, although the system is monostable, *c*(*κ*,*θ*_*i*_) is extremely small about the region of the former second steady state. This causes a very slow dynamic, which can be seen by tracking the normalized state variable *z*_3_(*t*), as indicated in the Figure. Only at *t*≈440 min *z*_3_(*t*) reaches an area where |*c*(*κ*,*θ*_*i*_)| becomes larger, which results in a subsequent fast convergence to the unique globally stable fixed point at the origin.

**Fig. 9:**
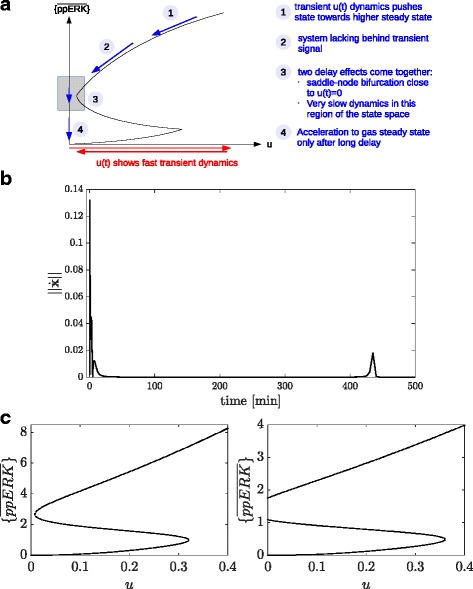
Combination of two delay mechanisms in quasi-bistable systems. **a** Scheme of a bifurcation diagram for a quasi-bistable system. The system is monostable for *u*=0 and has a saddle-node bifurcation *u* ^SNB^ close to *u*=0, where it becomes bistable. A sufficiently strong transient signal *u*(*t*) pushes the system state into the basin of attraction of the higher stable steady state (1). As long as the change in *u*(*t*) is not slow compared to the dynamics of the system, the system cannot be considered in quasi-steady state, and we observe a transient dynamics (2). When *u*(*t*) is almost back to 0, two delay effects lead to quasi-bistable behavior (3). First, the system remains in the upper stable steady state as long as *u*(*t*) is still above the saddle-node bifurcation. Second, for *u*(*t*)<*u* ^SNB^ the acceleration remains very small in this region of the state space. **b** Absolute value of the vector field along the model trajectory for the same model parameters that have been used in Fig. 8. **c** Two respresentative bifurcation diagrams for a quasi-bistable system belonging to class 2 of the classification scheme, and a bistable system belonging to class 1

Thus, taken together, this analysis suggests that quasi-bistability is caused by traversing a region in the state space in which $\dot x$ is extremely small, resulting in a very slow dynamics. The system is only accelerated towards it’s single steady state when the state of the system leaves this region. This makes the system behave as a bistable system for a long time span. This hypothesis was confirmed by a subsequent bifurcation analysis with some representative parameter sets for classes 1 and 2 of the classification scheme, as shown in Fig. 9. Figure 9 a illustrates the two effects that act together to delay the response of the system upon a transient stimulus. Figure 9 b shows the absolute value of the vector field ∥*f*(*x*(*t*,*θ*_*i*_))∥ of the same trajectory as in Fig. 8, which shows high values a few minutes after stimulation, followed by a long period where $\dot x(\theta _{i})$ is virtually zero, and a second peak at about *t*=440 min, where the trajectory is pushed towards the systems unique steady state. A comparison of bifurcation diagrams for representative parameter sets belonging to classes 2 (quasi-bistable) and 1 (bistable) is depicted in Fig. 9 c and shows that the difference between these two classes is actually ‘smooth’ in terms of changes in limit sets.

## Discussion

Our bifurcation analysis showed that the transition between the three classes for stability behavior classification (bistable, quasi-bistable and monostable) is actually smooth in terms of locations of bifurcation points and limit sets, and we expect the range of parameters in which quasi-bistability occurs to be rather small. This expectation was confirmed by a sensitivity analysis for the outcome of the simulation-based classification scheme (Additional file 8), in which we varied all model parameters independently one at a time about the maximum-a-posteriori estimator. The result shows that the appearance of quasi-bistability is highly sensitive to these variations for almost all parameters. Except for the phosphorylation rate of Raf (parameter $k_{1}^{+}$) and the threshold parameter *K* of the input function, which do not have any influence on the limit sets of the system for *u*=0, small variations of parameters induce switches to mono- or bistable systems, which is due to the fact that the location of the saddle-node bifurcation and hence the delay time are very sensitive to parameter changes. The fact that most of the samples fall into this seemingly small parameter range shows that the gradient of the posterior distribution must be rather high when varying parameters individually. This is indeed the case, since the fit quality rapidly drops at least for the NGF control experiment when switching from the quasi-bistable to the monostable range, and we observe a similar effect for the switch to the bistable range. Altogether, these results also indicate the existence of strong correlations between parameters in the posterior distribution.

We note here that the distribution of switching times of quasi-bistable trajectories of our inferred model partly disagrees with observed ERK activities for later time points. While most of the trajectories switch to their steady state between 60 and 120 min in our model, experiments in [37, 38] show that ERK activity is sustained for at least 2–3 h. Since our dataset only contained measurements for up to 60 min, this fact was not taken into account in the model calibration procedure. However, the response duration of the signaling cascade upon stimulation with NGF also seems to show a large variation and to depend in particular on the experimental protocol and distinct clonal PC-12 cell lines, as also stated in [37]. In [39] or [40], for example, MEK (MAP-2 kinase) and ERK activities are almost completely down to the basal level already after 2 h (see [39], Fig. 3 and [40], Fig. 9b, curve without treatment with TPA). Hence it is not completely clear how to describe the activity of the module quantitatively in order to enrich the model with knowledge on the long-term behavior. However, we started to investigate the effect of assuming different minimal switching times for ERK activity, which is illustrated in Additional file 9. As expected, filtering for trajectories that still have a substantial remaining activity after two and three hours, respectively, increases the ratio of bistable versus quasi-bistable trajectories, since only quasi-bistable trajectories are filtered out. Thus, we think that our model is generally able also to match the long-term behavior of the cascade.

Furthermore, we have not explicitly taken into account fluctuations in protein content, although we are aware that this is a major source of variability in cell populations. The total amounts of Raf, MEK and ERK do not explicitly appear any more in the rescaled and normalized model version that we used for our study, hence it is not possible to take absolute fluctuations into account. However, we investigated the effect of varying absolute concentrations by varying the coefficients *s*_*i*_, *i*=1,2,3 in a narrow range about its nominal values *s*_*i*_=1 and considered the sensitivity of bistability and quasi-bistability to these parameters. Exemplary results are shown for variations in ERK (*s*_3_) in Additional file 10. Figures for variations in *s*_1_ and *s*_2_ look very similar. Interestingly, the classification of trajectories seems to be very sensitive to these parameters. As can be seen, a moderate reduction in *s*_*i*_ is sufficient to destroy bistability and quasi-bistability almost completely, while bistability is strongly enhanced upon a slight increase in *s*_*i*_. This is a surprising result, since it is known that stochastic gene expression events can, for example, result in coefficients of variation of about 20-30% in the content of individual proteins [20, 28]. This raises the general question about reliability and robustness of decision processes under such variations. To our knowledge, minimal models for bistability, as used here, are often not robust with respect to such fluctuations and parameter variations, which might trigger further investigations in this direction.

## Conclusion

We presented a modeling study that focuses on mechanisms behind sustained responses of signaling pathways upon transient stimulation in PC-12 cells. The model is based on chemical reaction kinetics and was calibrated to a dataset of PC-12 cell lines that were stimulated with EGF and NGF in a control setting and under silencing perturbations. We used a sampling-based Bayesian approach for model calibration, and analyzed model predictions in terms of posterior predictive distributions, which provides complete information about remaining uncertainties. The model was validated by comparing model predictions of new scenarios to experimental data.

Interestingly, the system shows a sustained ERK activity profile upon NGF stimulation, while the response was transient in case of EGF stimulation. This phenomenon has been well-investigated experimentally and theoretically, and it is well believed that the observed sustained response is caused by a bistable system. Here we combine our statistical inference approach with steady state analysis to investigate mechanisms behind this sustained ERK response. Surprisingly, our results indicate that the probability for bistable behavior is far below the observed response, and thus suggest that it is not sufficient to concentrate analysis on steady states only. A simulation-based analysis of the phenomenon revealed the importance of quasi-bistability to shape ERK response. A system is said to be quasi-bistable, if it is monostable but able to maintain a state distinct from its steady state for a long time period. It is known that positive feedback can generally cause quasi-bistability [33], it has however not been shown that this is relevant for decision making in living systems.

For a biological system it might not make a difference at all whether the underlying system is indeed bistable or quasi-bistable, since the system probably acts as an integrator of a response, which starts to trigger further events as soon as a threshold has been reached. However, one has to be careful with the analysis of models for such mechanisms. Our results propose to consider, besides limit sets, also the transient behavior of a system when investigating processes such as switches, memory effects or decision making.

There is an ongoing debate about relations between ultrasensitivity and/or bistability in responses of single cells on the one hand and the occurrence of bimodality in heterogeneous cell populations on the other hand (see e.g. [9, 36]). It is clear that ultrasensitive and bistable systems can lead to bimodal responses, for example caused by variations in protein contents or stochastic fluctuations. An example that considers the role of mutual inhibition in a gene regulatory network for metastatic transitions and the appearance of stable subpopulations of genetically identical prometastatic cells is described in [41]. On the other hand, for the MAPK pathway it has been shown that bimodality can also emerge from graded single cell responses caused by a broad distribution of ERK pathway activation thresholds [36]. This example reveals that the relation between bistability and bimodality is actually more subtle than a simple one-to-one relation.

We had decided in this study to use a data-driven approach and to adapt model granularity to the data available for model calibration. This results of course in a very simplified model, and the situation in vivo is much more complex. Specific aspects regarding the MAPK signaling pathways are discussed in literature and have also partly been implemented in models. One of the most recent interesting studies investigates the role of feedbacks and their time scales by using pulse experiments on a single cell level (see also [42] for a commentary on this). Kocieniewski et al. [43], for example, focus on the role of the two different MEK isoforms and their contribution in the regulation of the ERK response. According to this study, response duration and amplitude are regulated by the ratio and the total amount of both isoforms, respectively. Moreover, localization of proteins and their regulation via scaffolding proteins, together with nucleoplasmic shuttling, is known to play a major role in the regulation of the pathway [9, 22, 28]. Cross-talk and interactions with other cellular pathways is another important aspect [9, 11], which is difficult to take into account in any modeling approach. However, it is an important and interesting question how single modules such as the MAPK signaling cascade behave embedded in a larger and more complex network. Several studies in recent years hint to the fact that network complexity is intimately linked to functional robustness, meaning that the network structure, and in particular interlinked feedback loops, contribute to a reliable performing of tasks in the presence of perturbations and noise [44–48].

A further critical point in our modeling study is the normalization of model outputs to a particular time point. This normalization was necessary since the dataset used for model calibration only provides relative information. Signals are given in arbitrary units, and the scaling factors are different for each antibody and can also vary across membranes. Thus normalization to a reference experiment is required to make measurements from different experiments comparable and is standard in representing biological data and for modeling [43]. This normalization, however, affects variances of observables, and precludes comparison with experiments where total protein levels matter, such as absolute heights of ppERK peaks under different conditions. Thus, including some information about total protein levels could highly enrich the modeling process in the future.

Finally, recently a new modeling approach, called ODE constraint mixture modeling, was introduced [49]. This approach combines advantages of mechanistic modeling approaches with statistical mixture models to describe heterogeneous cell populations. This framework allows to infer subpopulation structures and dynamics from single cell snapshot data. Since the data used here for model calibration represent only population averages, we did not explicitly take subpopulation structures into account. However, at least the dose response profiles of ERK after stimulation with NGF seem to consist of two or more subpopulations, which was also exploited to mimic the respective dose response curve. Thus, exploiting this framework is another interesting task for future investigations.

## Endnote

^1^ called in the following *circuits*, for consistency with graph-theoretic terminology.

## Additional files

Additional file 1 A Bayesian framework for ode model calibration. (PDF 276 kb)

Additional file 2 Model normalization procedure. (PDF 131 kb)

Additional file 3 Formulation of the posterior distribution. (PDF 130 kb)

Additional file 4 Details on the MCMC sampling procedure. (PDF 137 kb)

Additional file 5 Estimated marginal parameter distributions from the MCMC sample. (PDF 64.5 kb)

Additional file 6 Scatterplot matrix of a subset of the parameters from the MCMC sample. (PDF 8460 kb)

Additional file 7 Details on the classification scheme with the CBA. (PDF 104 kb)

Additional file 8 Sensitivity analysis of the simulation-based classification scheme. (PDF 175 kb)

Additional file 9 Simulation-based classification of sample trajectories with varying minimal switching times. (PDF 51.9 kb)

Additional file 10 Classification of trajectories from the MCMC sample with varying concentration of total ERK. (PDF 67.9 kb)

## Abbreviations

CBA: Circuit-breaking algorithm
MCMC: Markov chain Monte Carlo
PPD: Posterior predictive distribution
