# Supplementary material for: Sampling-based Bayesian approaches reveal the importance of quasi-bistable behavior in cellular decision processes on the example of the MAPK signaling pathway in PC-12 cell lines
Source: BMC Syst Biol. 2017 Jan 25;11:11. doi: 10.1186/s12918-017-0392-6 (PMC5267478; doi:10.1186/s12918-017-0392-6)
Supplement: Additional file 1 — A Bayesian framework for ode model calibration. (PDF 276 kb) [file 12918_2017_392_MOESM1_ESM.pdf]

Sampling-based Bayesian approaches reveal the importance of quasi-bistable behavior in cellular decision processes on the example of the MAPK signaling pathway in PC-12 cell lines

Antje Jensch, Caterina Thomaseth, Nicole E Radde

December 22, 2016

Additional file 1: A Bayesian framework for ode model calibration

In a Bayesian parameter estimation framework, every quantity-of-interest is described in terms of a probability distribution. This framework allows to propagate variability in the data to uncertainties in model predictions and is illustrated in Figure 1. The framework is initialized by encoding prior knowledge about parameters θ in a *prior probability distribution* $p(\theta)$, which is most often simply a uniform distribution within finite boundaries. Data are interpreted in this framework as samples from a parametrized stochastic process, which defines the likelihood function $p(y|\theta)$. In our framework, we employ *stochastically embedded* ODE models, i.e. we assume that the underlying process can be described in a deterministic way (the ODE model) and measurements are disrupted by measurement errors (the error model, also called noise model). The likelihood function is used to update our prior knowledge about model parameters and to transform it into a *posterior distribution* $p(\theta|y)$, which is a distribution of the model parameters conditional on the data. This is obtained via exploiting Bayes' Theorem. This posterior distribution can in principle be transformed into *posterior predictive distributions* $p(\tilde{y}|y)$ for any quantity-of-interest $\tilde{y}$, like e.g. marginals of individual parameters, model states, event times, or discrete features emerging from the model's behavior such as quasi-bistability.

In the particular framework of ODE model parameter estimation, we face the problem that the posterior distribution is not available in closed form. Thus, it is investigated via generating respresentative samples, which is realized via constructing a Markov chain that converges to the desired target distribution.

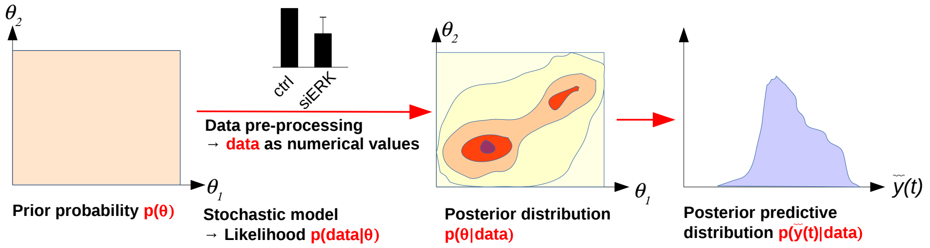

Figure 1: Schematic of a Bayesian learning framework.

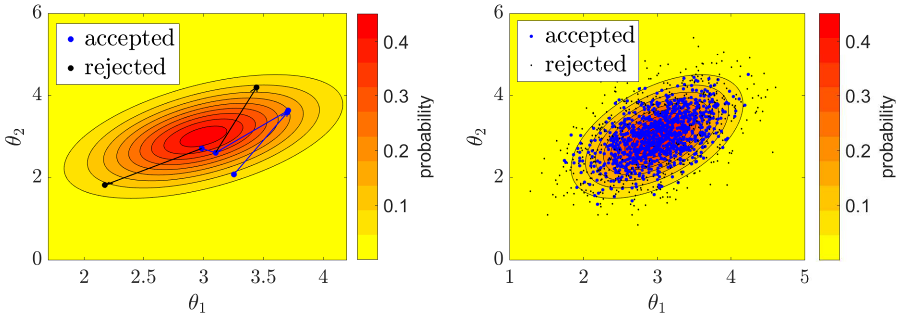

Figure 2: Schematic of Markov chain Monte Carlo sampling.

There are numerous algorithms available for this (for more details and historical work we refer to [1, 2, 3]). The working principle of such an MCMC algorithm is shown in Figure 2. Situated at θ, the Markov chain proposes a new parameter set θ', which is accepted with a probability that takes the ratio of the values of the target density at θ and θ' into account (Figure 2 left). If the chain is converged, the set of accepted samples represent the target distribution (Figure 2 right).

References

[1] Gelman A, Carlin JB, Stern HS, Rubin DB. Bayesian Data Analysis. 2nd edition, Texts in Statistical Science, Chapman & Hall/CRC 2006.

[2] Hastings WK. Monte Carlo sampling methods using Markov chains and their applications. Biometrika 1970; 57(1): 97–109.

[3] Metropolis N et al. Equation of state calculations by fast computing machines. J Chem Phys 1953; 21(6): 1087–92.
