# Supplementary material for: Sampling-based Bayesian approaches reveal the importance of quasi-bistable behavior in cellular decision processes on the example of the MAPK signaling pathway in PC-12 cell lines
Source: BMC Syst Biol. 2017 Jan 25;11:11. doi: 10.1186/s12918-017-0392-6 (PMC5267478; doi:10.1186/s12918-017-0392-6)
Supplement: Additional file 2 — Model normalization procedure. (PDF 131 kb) [file 12918_2017_392_MOESM2_ESM.pdf]

Sampling-based Bayesian approaches reveal the importance of quasi-bistable behavior in cellular decision processes on the example of the MAPK signaling pathway in PC-12 cell lines

Antje Jensch, Caterina Thomaseth, Nicole E Radde

October 18, 2016

Additional file 2: **Model normalization procedure**

We start with the model version shown in Fig 3B in the main manuscript

$$
\begin{aligned}
\dot{\text{pRaf}} &= k_1^+(\text{Raf}_{\text{TOT}}s_1 - \text{pRaf})u(t) - k_1^-\text{pRaf} + \\
&\quad + fn\left[-k_{Fn}\text{ppERKpRaf}\right] + \\
&\quad + fp\left[k_{Fp}\frac{\text{ppERK}^5}{\text{ppERK}^5 + g^5}(\text{Raf}_{\text{TOT}}s_1 - \text{pRaf})\right] \\
\dot{\text{ppMEK}} &= k_2^+(\text{MEK}_{\text{TOT}}s_2 - \text{ppMEK})\text{pRaf} - k_2^-\text{ppMEK} \\
\dot{\text{pERK}} &= k_3^+(\text{ERK}_{\text{TOT}}s_3 - \text{pERK} - \text{ppERK})\text{ppMEK} + \\
&\quad + k_4^-\text{ppERK} - (k_3^- + k_4^+\text{ppMEK})\text{pERK} \\
\dot{\text{ppERK}} &= k_4^+\text{pERKppMEK} - k_4^-\text{ppERK} \\
u(t) &= \begin{cases} 0 & t < 0 \\ 1 - \dfrac{t^3}{t^3 + K^3} & t \geq 0. \end{cases}
\end{aligned}
$$

In order to compare this model to the data in [1], variables have to be rescaled and normalized to the same reference experiment as in [1]. The light signals detected in the Western blots were normalized to the signals of the respective total proteins, such that the experimental values represent measures that are proportional to the fractions of phosphorylated proteins. Following this line of argumentation, we rescale the variables of the model accordingly, by defining the dimensionless state variables as

$$
\begin{aligned}
x_1 &= \alpha_1 \cdot \frac{\text{pRaf}}{\text{Raf}_{\text{TOT}} \cdot s_1} \\
x_2 &= \alpha_2 \cdot \frac{\text{ppMEK}}{\text{MEK}_{\text{TOT}} \cdot s_2} \\
x_3 &= \alpha_3 \cdot \frac{\text{pERK}}{\text{ERK}_{\text{TOT}} \cdot s_3} \\
x_4 &= \alpha_4 \cdot \frac{\text{ppERK}}{\text{ERK}_{\text{TOT}} \cdot s_3}.
\end{aligned}
$$

The transformed system in terms of these new variables reads

$$
\begin{aligned}
\dot{x}_1 &= \mathbf{k_1^+}(\alpha_1 - x_1)u - \mathbf{k_1^-}x_1 + \\
&\quad + fn\left[-\mathbf{\tilde{k}_{Fn}}\frac{1}{\alpha_4}s_3 x_1 x_4\right] + fp\left[\mathbf{k_{Fp}}\frac{x_4^5}{x_4^5 + \left(\frac{\mathbf{\tilde{g}}\alpha_4}{s_3}\right)^5}(\alpha_1 - x_1)\right] \\
\dot{x}_2 &= \mathbf{\tilde{k}_2^+}(\alpha_2 - x_2)s_1\frac{1}{\alpha_1}x_1 - \mathbf{k_2^-}x_2 \\
\dot{x}_3 &= \mathbf{\tilde{k}_3^+}(1 - x_3 - \frac{1}{\alpha_4}x_4)s_2\frac{1}{\alpha_2}x_2 + \mathbf{k_4^-}\frac{1}{\alpha_4}x_4 - \mathbf{k_3^-}x_3 - \mathbf{\tilde{k}_4^+}s_2\frac{1}{\alpha_2}x_3 x_2 \\
\dot{x}_4 &= \mathbf{\tilde{k}_4^+}s_2\frac{\alpha_4}{\alpha_2}x_3 x_2 - \mathbf{k_4^-}x_4.
\end{aligned}
$$

Here, bold parameters are unknown and have to be estimated. Gray parameters specify the experimental condition. We have set $\alpha_3 = 1$ w.l.o.g., since pERK was not quantified experimentally. Rescaling of parameters is given by the transformations

$$
\begin{aligned}
\tilde{g} &= \frac{g}{\text{ERK}_{\text{TOT}}} \\
\tilde{k}_{Fn} &= k_{Fn}\text{ERK}_{\text{TOT}} \\
\tilde{k}_2^+ &= k_2^+\text{Raf}_{\text{TOT}} \\
\tilde{k}_3^+ &= k_3^+\text{MEK}_{\text{TOT}} \\
\tilde{k}_4^+ &= k_4^+\text{MEK}_{\text{TOT}}.
\end{aligned}
$$

In the following, to keep the notation as simple as possible, we will neglect the tilde for the rescaled parameters, and therefore consider the obtained ODE model $\dot{x} = f(x, \theta), x \in \mathbb{R}_+^4$, with parameter vector $\theta \in \mathbb{R}_+^{12}$ given by

$$\theta = (k_1^+, k_2^+, k_3^+, k_4^+, k_1^-, k_2^-, k_3^-, k_4^-, k_{Fn}, k_{Fp}, g, K).$$

The coefficients α_i account for the effect of different antibodies and their binding affinities in the Western blot measurements. These are furthermore additionally dependent on the particular experimental conditions and the specialties of the membranes. Thus, in order to enable a comparison across experiments on different membranes, Western blot data are usually additionally normalized to a reference condition. Following the data in Santos et al., we used the states at $t^* = 5$ min as the reference condition for each individual protein for this purpose, and the model outputs were normalized accordingly:

$$
\begin{aligned}
z_1(t) &= \frac{x_1(t)}{x_1(t^* = 5\text{min})} = \frac{\text{pRaf}(t)}{\text{pRaf}(t^* = 5\text{min})} \\
z_2(t) &= \frac{x_2(t)}{x_2(t^* = 5\text{min})} = \frac{\text{ppMEK}(t)}{\text{ppMEK}(t^* = 5\text{min})} \\
z_3(t) &= \frac{x_4(t)}{x_4(t^* = 5\text{min})} = \frac{\text{ppERK}(t)}{\text{ppERK}(t^* = 5\text{min})}.
\end{aligned}
$$

These output variables are independent of the scaling factors α_i, yet these are needed to simulate the model output during the optimization. Here we chose the interval $[0, 4]$ to sample the alphas during the MCMC procedure.

References

[1] Santos SDM, Verveer PJ, Bastiaens PIH. Growth factor-induced MAPK network topology shapes ERK response determining PC-12 cell fate. Nat Cell Biol. 2007; 9(3): 324–30.
