# Supplementary material for: Sampling-based Bayesian approaches reveal the importance of quasi-bistable behavior in cellular decision processes on the example of the MAPK signaling pathway in PC-12 cell lines
Source: BMC Syst Biol. 2017 Jan 25;11:11. doi: 10.1186/s12918-017-0392-6 (PMC5267478; doi:10.1186/s12918-017-0392-6)
Supplement: Additional file 3 — Formulation of the posterior distribution. (PDF 130 kb) [file 12918_2017_392_MOESM3_ESM.pdf]

Sampling-based Bayesian approaches reveal the importance of quasi-bistable behavior in cellular decision processes on the example of the MAPK signaling pathway in PC-12 cell lines

Antje Jensch, Caterina Thomaseth, Nicole E Radde

December 22, 2016

Additional file 3:
Formulation of the posterior distribution

For our Bayesian parameter estimation framework we need to formulate the posterior distribution,

$$p(\theta|y) = \frac{l_y(\theta)p(\theta)}{p(y)}. \tag{1}$$

We will first define the likelihood function $l_y(\theta)$. Therefore, we assume log normally distributed error models for each individual measurement,

$$\tilde{Y}_i(t_k) \sim \log N(\log x_i(t_k), \sigma_{ik}^2),$$

which leads to

$$Y_i(t_k) \sim \log N(\log x_i(t_k) - \log x_i(t^*), \sigma_{ik}^2 + \sigma_{i*}^2)$$

for the normalized data, where σ_{i*}^2 denotes the error of the reference experiment for protein i.

For the global response coefficients R_{ij}, $i, j = 1, 2, 3$ we take the values in [1], which consist of four replicates. As described in the main manuscript, the global response coefficients (GRC) are defined as

$$R_{ij} = 2\frac{\partial \ln(v_i)}{\partial \ln(p_j)} \approx 2\frac{(\bar{v}_i^{(s_j)} - \bar{v}_i^{(c)})}{(\bar{v}_i^{(s_j)} + \bar{v}_i^{(c)})},$$

where v_1 = pRaf, v_2 = ppMEK, v_3 = ppERK. The variables $\bar{v}_i^{(s_j)}$ and $\bar{v}_i^{(c)}$ denote the (quasi) steady state activities of component i in the case of silencing of component j and in the control case, respectively. In order to approximate these steady state values, measurement time points were set to $t_{\text{GRC}}^{\text{EGF}} = 5$ min and $t_{\text{GRC}}^{\text{NGF}} \in \{5, 15\}$ min (for more details we refer to [1] and references therein). Hence

$$R_{ij} \approx R_{ij}(t_k) \approx 2\frac{(v_i^{(s_j)}(t_k) - v_i^{(c)}(t_k))}{(v_i^{(s_j)}(t_k) + v_i^{(c)}(t_k))},$$

where $v_i^{(s_j)}(t_k)$ and $v_i^{(c)}(t_k)$ denote the activities of component i at time point t_k in the case of silencing of component j and in the control case, respectively.

The table in Fig 2 in the main manuscript lists estimates $\widehat{\mathbb{E}}(R_{ij}(t_k))$ and $\widehat{\sigma}(R_{ij}(t_k))$ extracted from the data in [1]. To remain consistent with our hypothesis of log normal distributions for the (normalized) Western blot signals, we decided to use these estimates to obtain respective estimates for the parameters of the quantity

$$z'_{ij}(t_k) := \frac{v_i^{(s_j)}(t_k)}{v_i^{(c)}(t_k)},$$

since corresponding measurement values $Y'_{ij}(t_k)$ also follow a log normal distribution. Therefore, we resolved $R_{ij}(t_k)$ for $z'_{ij}(t_k)$ to get

$$z'_{ij}(t_k) = \frac{2 + R_{ij}(t_k)}{2 - R_{ij}(t_k)}.$$

According to this, estimates for the parameters of the log normal distribution of $Y'_i(t_k)$ were set to

$$\widehat{\mathbb{E}}(Y'_{ij}(t_k)) = \frac{2 + \widehat{\mathbb{E}}(R_{ij}(t_k))}{2 - \widehat{\mathbb{E}}(R_{ij}(t_k))}$$

and

$$\begin{aligned}\widehat{\sigma}^2(Y'_{ij}(t_k)) &= \left| \frac{\partial z'_{ij}(t_k)}{\partial R_{ij}(t_k)} \right| \; \widehat{\sigma}(R_{ij}(t_k)) \\ &= \frac{4}{(2 - \widehat{\mathbb{E}}(R_{ij}(t_k)))^2} \widehat{\sigma}(R_{ij}(t_k)).\end{aligned}$$

In summary, the resulting likelihood function reads:

$$l_{\log y}(\theta) = \prod_{m} \prod_{i=1}^{3} \left(\prod_{t_k} \frac{1}{\sqrt{2\pi\sigma_{imk}^2}} \exp\left[-\frac{1}{2} \left(\frac{\log z_i^m(t_k, \theta) - \log y_i^m(t_k)}{\sigma_{imk}} \right)^2 \right] \right) \times$$
$$\prod_{j=1}^{3} \prod_{t_{\text{GRC}}^m} \left(\frac{1}{\sqrt{2\pi\widehat{\sigma}_{ijm}^2(t_{\text{GRC}}^m)}} \exp\left[-\frac{1}{2} \left(\frac{\log \widehat{\mathbb{E}}(Y'_{ij}(t_{\text{GRC}}^m)) - \log z'_{ij}(t_{\text{GRC}}^m, \theta)}{\widehat{\sigma}_{ijm}^2(t_{\text{GRC}}^m)} \right)^2 \right] \right)$$

Here, $m \in \{\text{EGF,NGF}\}$ denote experiments with different growth factors, the indices $i = 1, 2, 3$ and $j = 1, 2, 3$ enumerate the three output variables z_i and the three silencing experiments siRaf, siMEK and siERK, respectively. The time points $t_k \in \{10, 15, 30, 60\}$ min refer to the meausurement time points in the control experiments, and $t_{\text{GRC}}^{\text{EGF}} = 5$ min for the time point that is used to determine the global response coefficients in case of stimulation with EGF and $t_{\text{GRC}}^{\text{NGF}} \in \{5, 15\}$ min for the two time points used in the respective NGF experiments. We note here that $t_k = 5$ min does not appear in the likelihood function, since measurements at this point were used as reference experiments.

Since a priori nothing was known about the values of the parameters

$$\theta = (k_1^+, k_2^+, k_3^+, k_4^+, k_1^-, k_2^-, k_3^-, k_4^-, k_{Fn}, k_{Fp}, g, K) \in \mathbb{R}_+^{12},$$

we decided to use almost non-informative prior distributions. This was done by assuming uniform distributions on the logarithmic scale for all parameters but K in order to allow for covering several orders of magnitude for these parameters. For details on the choice of the prior boundaries and the optimization and subsequent sampling we refer to Additional file 4.

References

[1] Santos SDM, Verveer PJ, Bastiaens PIH. Growth factor-induced MAPK network topology shapes ERK response determining PC-12 cell fate. Nat Cell Biol. 2007; 9(3): 324–30.
