# Supplementary material for: Sampling-based Bayesian approaches reveal the importance of quasi-bistable behavior in cellular decision processes on the example of the MAPK signaling pathway in PC-12 cell lines
Source: BMC Syst Biol. 2017 Jan 25;11:11. doi: 10.1186/s12918-017-0392-6 (PMC5267478; doi:10.1186/s12918-017-0392-6)
Supplement: Additional file 4 — Details on the MCMC sampling procedure. (PDF 137 kb) [file 12918_2017_392_MOESM4_ESM.pdf]

Sampling-based Bayesian approaches reveal the importance of quasi-bistable behavior in cellular decision processes on the example of the MAPK signaling pathway in PC-12 cell lines

Antje Jensch, Caterina Thomaseth, Nicole E Radde

October 18, 2016

Additional file 4: **Details on the MCMC sampling procedure**

In order to sample from the posterior distribution described in Additional file 3, all numerical calculations were run on `MATLAB R2014b` (64 bit). The model and data were managed using the toolboxes `SBPD` and `SBTOOLBOX2`. `SBTOOLBOX2` with the CVODE integrator from `SUNDIALS` was employed for the integration of the ODE system. Absolute and relative error tolerances of the integrator were set to `options.abstol=1e-10` and `options.reltol=1e-10`.

In the first step we intended to find good starting values for the Markov chains and appropriate boundaries for the parameter's prior distributions. As described, all parameters except K were sampled in the log space, to cover several orders of magnitudes. Since K describes the decay or switching time in the input, which is expected from the EGF control experiments to lie approximately between 5 and below 10 minutes, we used a uniform distribution with fixed boundaries $[4, 8]$ min for this parameter directly. The boundaries for the other distributions were set heuristically via a trial and error procedure. Therefore, in a first step we optimized the posterior distribution several times with different prior boundaries and adapted the boundaries accordingly to ensure that parameter regions with very high likelihood values are not truncated by the prior distribution. Maximization of $p(\theta|y)$ was done by minimizing $-\log p(\theta|y)$ using the Matlab built-in function `fmincon`. Tolerances on the constraint violation and function value were set to `OPTIONSfmincon.TolFun=1e-6` and `OPTIONSfmincon.TolCon=1e-6`, respectively. To account for possible multiple local minima a multistart algorithm with uniformly distributed initial values was used.

Equipped with a convenient estimate $\widehat{\theta}^{\mathrm{MAP}}$ from this procedure (listed in Table 1), boundaries were set to $[10^{\widehat{\theta}^{\mathrm{MAP}}-2}, 10^{\widehat{\theta}^{\mathrm{MAP}}+2}]$ for subsequent MCMC sampling [1].

For the implementation the `mcmcstat` toolbox with the method option 'DRAM' was used. To achieve convergence a warm-up period of $5 \cdot 10^5$ samples was carried out prior to the sampling of a parameter chain of length $3 \cdot 10^6$. Four independent chains were initialized using as starting points different parameter estimates with small objective function values. Convergence for the overall chain was assessed with the Gelman-Rubin-Brooks diagnostic using the function `mpsrf`, which returns a potential scale reduction factor R. For testing of

Table 1: Estimated MAP parameter values.

θ	$\log k_1^+$	$\log k_2^+$	$\log k_3^+$	$\log k_4^+$	$\log k_1^-$	$\log k_2^-$
$\hat{\theta}^{\text{MLE}}$	-5.7324	7.3475	7.8110	2.3365	-0.0865	6.2055
θ	$\log k_3^-$	$\log k_4^-$	$\log k_{Fn}$	$\log k_{Fp}$	$\log g$	K
$\hat{\theta}^{\text{MLE}}$	6.8132	-0.4295	17,8312	-5.9037	-5.8563	5.6202

the individual chains the Geweke method was applied. Both diagnostics are implemented in `mcmcstat` [2].

The mean acceptance rate over the four chains was 11% in a first sampling trial. Convergence diagnostics showed $R = 1.0264$ for the Gelman-Rubin-Brooks method, but bad p-values for two chains with the Geweke method. To improve the sample quality a second sampling was carried out with initial parameters chosen from a sub-sample of the first run. The acceptance rate was improved to 20%. All chains passed the convergence test with a p-value of at least 0.8. Overall chain testing resulted in an improved value of $R = 1.0051$.

The estimates of the marginal distributions of the parameters from this second run are shown in Additional file 5. Highest and lowest indicated values on the abscissa correspond to lower and upper boundaries of the respective prior distributions. Estimates of the MAPs and the means are indicated by dashed gray lines and gray lines, respectively. It can be seen that most of these 1D marginals show a large variance, indicating that these are only vaguely defined, and that the data do not contain much information about these individual parameters. This is indeed not unusual in case of quantitative models and only few datapoints with high measurement noises. Only the distributions of the parameters k_1^- and k_4^- have significantly lower variances than the respective prior distributions, indicating a high sensitivity of the model output on these parameters.

References

[1] Haario H, Laine M, Mira A, Saksman E. DRAM: Efficient adaptive MCMC. Statistics and Computing. 2006. vol. 16, p. 339–354.

[2] Brooks S, Roberts G. Assessing Convergence of Markov Chain Monte Carlo Algorithms. Statistics and Computing. 1998. vol. 8, p. 319–335.
