# Supplementary material for: Sampling-based Bayesian approaches reveal the importance of quasi-bistable behavior in cellular decision processes on the example of the MAPK signaling pathway in PC-12 cell lines
Source: BMC Syst Biol. 2017 Jan 25;11:11. doi: 10.1186/s12918-017-0392-6 (PMC5267478; doi:10.1186/s12918-017-0392-6)
Supplement: Additional file 5 — Estimated marginal parameter distributions from the MCMC sample. (PDF 64.5 kb) [file 12918_2017_392_MOESM5_ESM.pdf]

Sampling-based Bayesian approaches reveal the importance of quasi-bistable behavior in cellular decision processes on the example of the MAPK signaling pathway in PC-12 cell lines

Antje Jensch, Caterina Thomaseth, Nicole E Radde

October 18, 2016

Additional file 5:
Estimated marginal parameter distributions from the MCMC sample

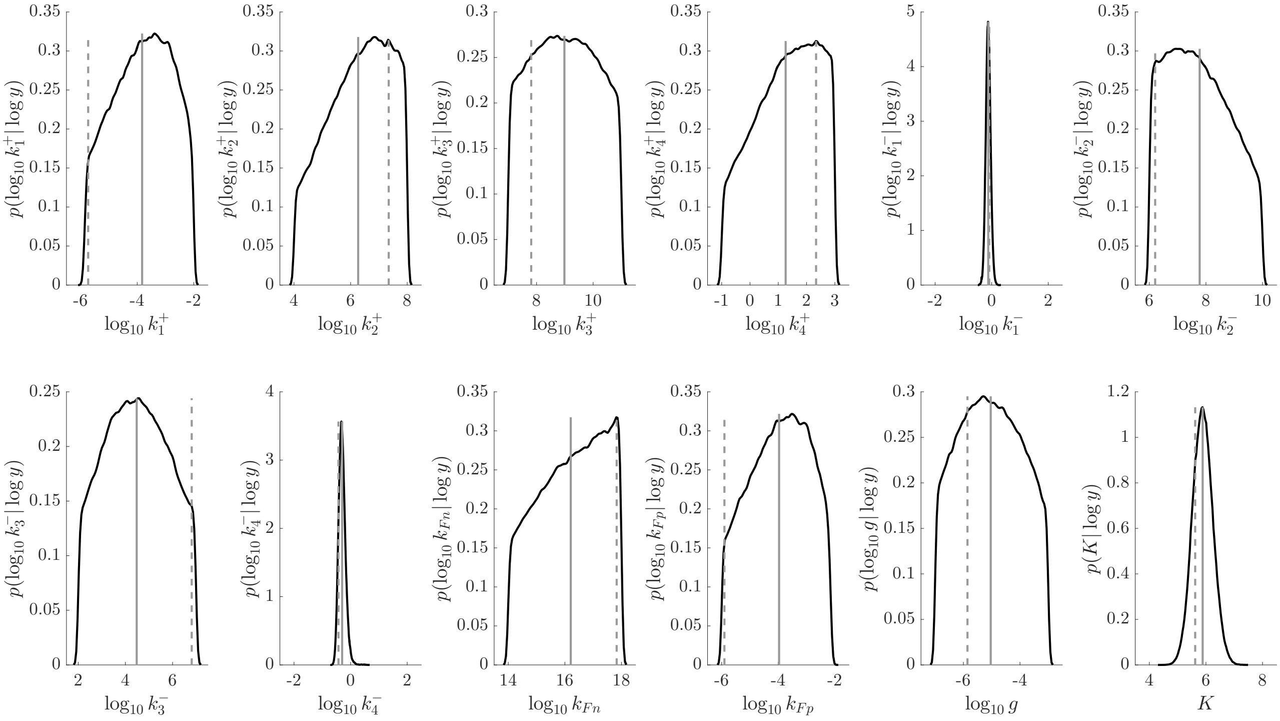

Figure 1: Kernel density estimates from the MCMC parameter samples of the marginal parameter distributions. Estimates of the MAPs and the means are indicated by dashed grey lines and grey lines, respectively.
