# Supplementary material for: Sampling-based Bayesian approaches reveal the importance of quasi-bistable behavior in cellular decision processes on the example of the MAPK signaling pathway in PC-12 cell lines
Source: BMC Syst Biol. 2017 Jan 25;11:11. doi: 10.1186/s12918-017-0392-6 (PMC5267478; doi:10.1186/s12918-017-0392-6)
Supplement: Additional file 6 — Scatterplot matrix of a subset of the parameters from the MCMC sample. (PDF 8460 kb) [file 12918_2017_392_MOESM6_ESM.pdf]

Sampling-based Bayesian approaches reveal the importance of quasi-bistable behavior in cellular decision processes on the example of the MAPK signaling pathway in PC-12 cell lines

Antje Jensch, Caterina Thomaseth, Nicole E Radde

October 18, 2016

Additional file 6:
Scatterplot matrix of a subset of the parameters from the MCMC sample

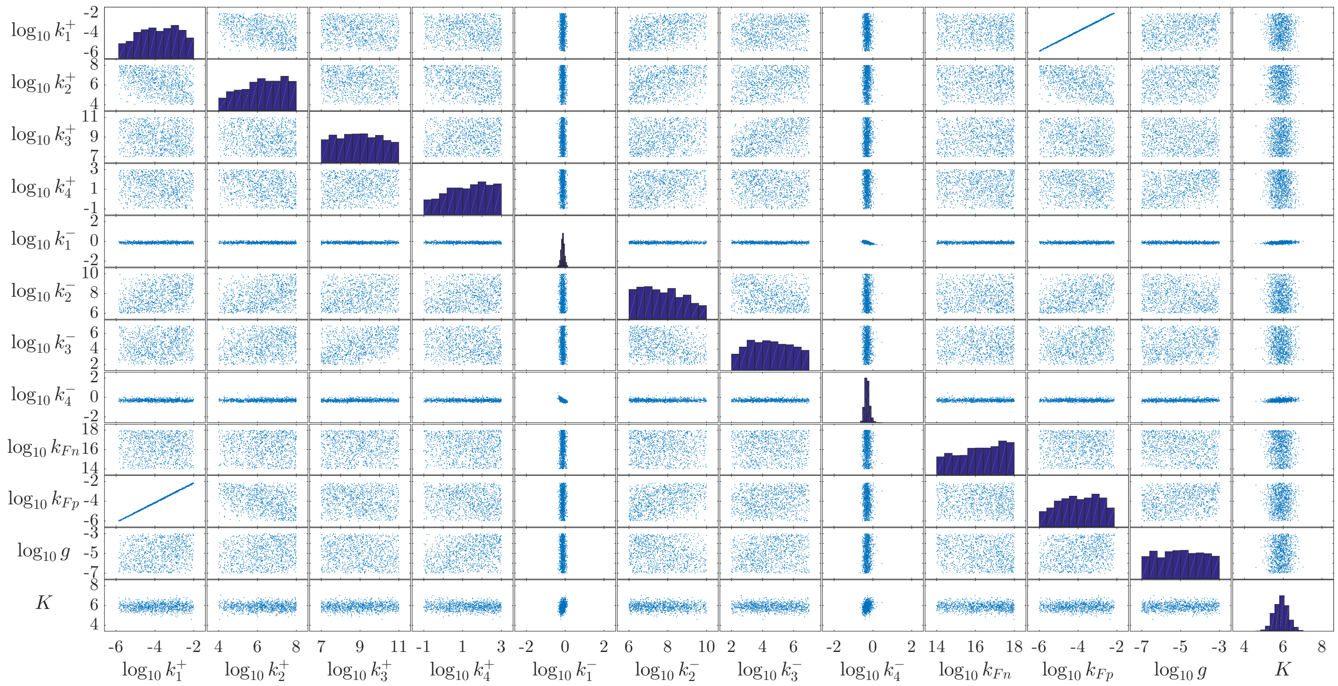

Figure 1: 2D scatter plot matrix for the parameters with histograms on the diagonal.
