# Supplementary material for: Sampling-based Bayesian approaches reveal the importance of quasi-bistable behavior in cellular decision processes on the example of the MAPK signaling pathway in PC-12 cell lines
Source: BMC Syst Biol. 2017 Jan 25;11:11. doi: 10.1186/s12918-017-0392-6 (PMC5267478; doi:10.1186/s12918-017-0392-6)
Supplement: Additional file 7 — Details on the classification scheme with the CBA. (PDF 104 kb) [file 12918_2017_392_MOESM7_ESM.pdf]

Sampling-based Bayesian approaches reveal the importance of quasi-bistable behavior in cellular decision processes on the example of the MAPK signaling pathway in PC-12 cell lines

Antje Jensch, Caterina Thomaseth, Nicole E Radde

October 18, 2016

Additional file 7: **Details on the classification scheme with the CBA**

In the main text we presented the steps of the circuit-breaking algorithm applied to our network model, represented schematically in Fig 6, Subfigs A-C. The final step of the algorithm, needed to obtain all steady-state coordinates for all variables of the system, requires the calculation of the zeros of the circuit-characteristics $c(\kappa, \theta_i)$ for all sample points θ_i. This condition is given by Equation (7) in the main manuscript.

For the rescaled and normalized model (see Additional file 2)

$$\begin{aligned}
\dot{x}_1 &= k_1^+(\alpha_1 - x_1)u - k_1^- x_1 + \\
&\quad + fn\left[-k_{Fn}\frac{1}{\alpha_4}s_3 x_1 x_4\right] + fp\left[k_{Fp}\frac{x_4^m}{x_4^m + \left(\dfrac{\tilde{g}\alpha_4}{s_3}\right)^m}(\alpha_1 - x_1)\right] && \text{(1a)}\\
\dot{x}_2 &= \tilde{k}_2^+(\alpha_2 - x_2)s_1\frac{1}{\alpha_1}x_1 - k_2^- x_2 && \text{(1b)}\\
\dot{x}_3 &= \tilde{k}_3^+(1 - x_3 - \frac{1}{\alpha_4}x_4)s_2\frac{1}{\alpha_2}x_2 + k_4^-\frac{1}{\alpha_4}x_4 - k_3^- x_3 - \tilde{k}_4^+ s_2\frac{1}{\alpha_2}x_3 x_2 && \text{(1c)}\\
\dot{x}_4 &= \tilde{k}_4^+ s_2\frac{\alpha_4}{\alpha_2}x_3 x_2 - k_4^- x_4, && \text{(1d)}
\end{aligned}$$

this translates into finding the intersection of two one-dimensional functions of κ in the particular example:

$$k_4^-\kappa = k_4^+ s_2\frac{\alpha_4}{\alpha_2}\bar{x}_2(\kappa, \theta_i)\bar{x}_3(\kappa, \theta_i), \tag{2}$$

as can be easily verified by looking at equation (1d) of the ODE model (1). The steady states of the other three state variables as function of κ and of the

model parameters are given by the following expressions:

$$\bar{x}_1(\kappa,\theta_i) = \alpha_1 \cdot \frac{k_{Fp} h(\kappa,\theta_i)}{k_1^- + k_{Fp} h(\kappa,\theta_i)} \tag{3a}$$

$$\bar{x}_2(\kappa,\theta_i) = \alpha_2 \cdot \frac{k_2^+ \dfrac{s_1}{\alpha_1} \bar{x}_1(\kappa,\theta_i)}{k_2^- + k_2^+ \dfrac{s_1}{\alpha_1} \bar{x}_1(\kappa,\theta_i)} \tag{3b}$$

$$\bar{x}_3(\kappa,\theta_i) = \frac{\dfrac{k_4^-}{\alpha_4}\kappa + k_3^+ \left(1 - \dfrac{\kappa}{\alpha_4}\right) \dfrac{s_2}{\alpha_2} \bar{x}_2(\kappa,\theta_i)}{k_3^- + \left(k_3^+ + k_4^+\right) \dfrac{s_2}{\alpha_2} \bar{x}_2(\kappa,\theta_i)}. \tag{3c}$$

In equation (3a) the function $h(\kappa,\theta_i)$ represents the Hill function

$$h(\kappa,\theta_i) = \frac{\kappa^m}{\kappa^m + (g\alpha_4/s_3)^m}. \tag{4}$$

Equation (2) is solved numerically. The set of solutions $\{\bar{\kappa}\}$ corresponds to the steady state coordinates of variable z_3.
