# Supplementary material for: Sampling-based Bayesian approaches reveal the importance of quasi-bistable behavior in cellular decision processes on the example of the MAPK signaling pathway in PC-12 cell lines
Source: BMC Syst Biol. 2017 Jan 25;11:11. doi: 10.1186/s12918-017-0392-6 (PMC5267478; doi:10.1186/s12918-017-0392-6)
Supplement: Additional file 8 — Sensitivity analysis of the simulation-based classification scheme. (PDF 175 kb) [file 12918_2017_392_MOESM8_ESM.pdf]

Sampling-based Bayesian approaches reveal the importance of quasi-bistable behavior in cellular decision processes on the example of the MAPK signaling pathway in PC-12 cell lines

Antje Jensch, Caterina Thomaseth, Nicole E Radde

October 18, 2016

Additional file 8:
Sensitivity analysis of the simulation-based classification scheme

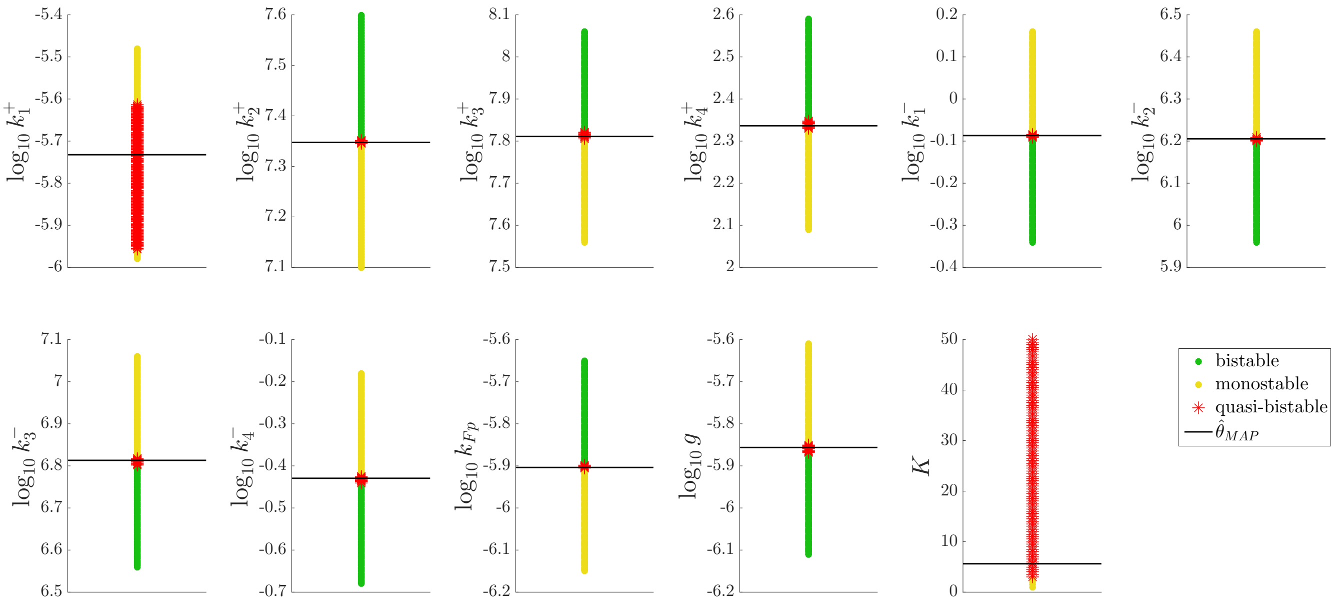

Figure 1: Model parameters were varied independently each at a time about the maximum-a-posteriori estimator. The simulation-based classification scheme was conducted repeatedly for these variations. Except for the parameters k_1^+ and K, which do not influence the limit sets of the system for $u = 0$, classification is highly sensitive to parameter variations.
