# Supplementary material for: Sampling-based Bayesian approaches reveal the importance of quasi-bistable behavior in cellular decision processes on the example of the MAPK signaling pathway in PC-12 cell lines
Source: BMC Syst Biol. 2017 Jan 25;11:11. doi: 10.1186/s12918-017-0392-6 (PMC5267478; doi:10.1186/s12918-017-0392-6)
Supplement: Additional file 9 — Simulation-based classification of sample trajectories with varying minimal switching times. (PDF 51.9 kb) [file 12918_2017_392_MOESM9_ESM.pdf]

Sampling-based Bayesian approaches reveal the importance of quasi-bistable behavior in cellular decision processes on the example of the MAPK signaling pathway in PC-12 cell lines

Antje Jensch, Caterina Thomaseth, Nicole E Radde

October 18, 2016

Additional file 9:
Simulation-based classification of sample trajectories with varying minimal switching times

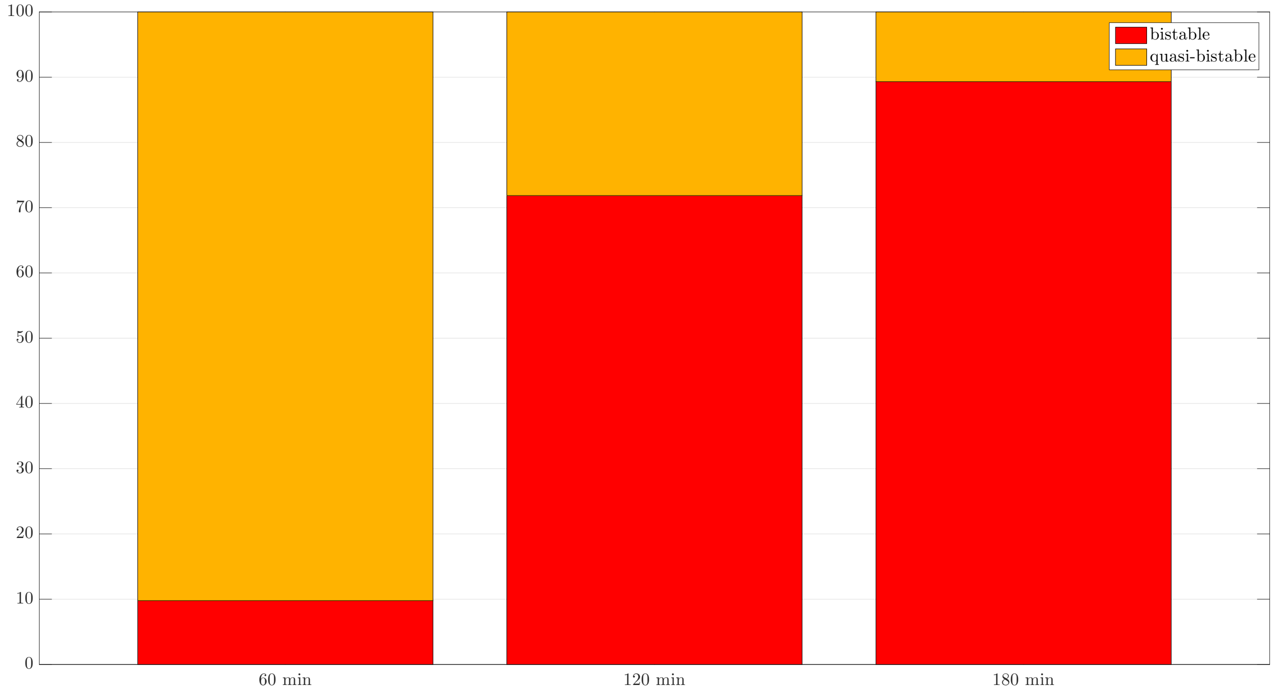

Figure 1: The 60 min case represents the full MCMC sample. For the 120 (180) min case all quasi-bistable trajectories that switch between 60 and 120 (180) min were filtered out.
