# Supplementary material for: Sampling-based Bayesian approaches reveal the importance of quasi-bistable behavior in cellular decision processes on the example of the MAPK signaling pathway in PC-12 cell lines
Source: BMC Syst Biol. 2017 Jan 25;11:11. doi: 10.1186/s12918-017-0392-6 (PMC5267478; doi:10.1186/s12918-017-0392-6)
Supplement: Additional file 10 — Classification of trajectories from the MCMC sample with varying concentration of total ERK. (PDF 67.9 kb) [file 12918_2017_392_MOESM10_ESM.pdf]

Sampling-based Bayesian approaches reveal the importance of quasi-bistable behavior in cellular decision processes on the example of the MAPK signaling pathway in PC-12 cell lines

Antje Jensch, Caterina Thomaseth, Nicole E Radde

October 18, 2016

Additional file 10:
Classification of sample trajectories with varying total ERK concentration

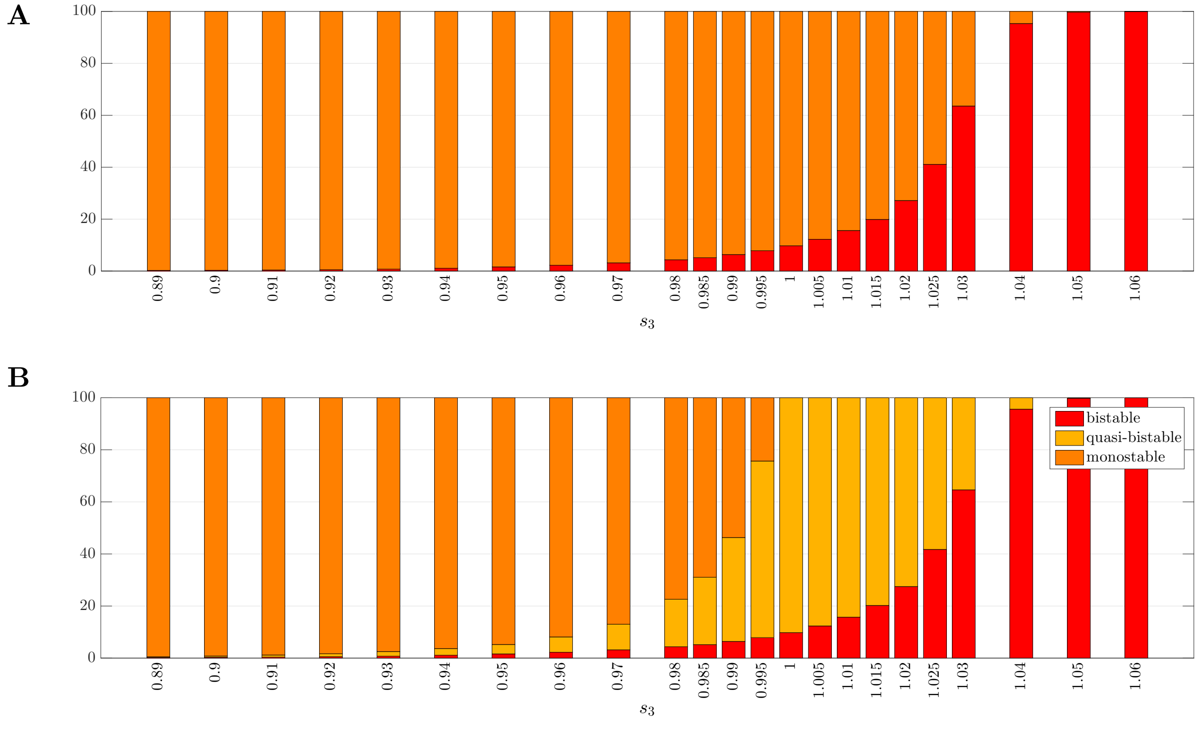

Figure 1: A. Steady state analysis via the CBA and classification into monostable and bistable trajectories. B. Simulation-based classification into bistable, quasi-bistable and monostable trajectories.
